# Early-stage environmental impact forecasting of chemicals and processes with machine learning and data analytics tools

**DOI:** 10.1007/s10098-026-03479-8

**Published:** 2026-04-04

**Authors:** Harriet Dufie Appiah, Matthew Conway, Jahnvi Patel, Marcella McMahon, Robert Hesketh, Kirti M. Yenkie

**Affiliations:** Department of Chemical Engineering, Rowan University, Glassboro, NJ USA

**Keywords:** Life cycle assessment, Machine learning, Artificial neural network, Extreme gradient boosting

## Abstract

Early-stage environmental evaluation of chemicals and process technologies is often constrained by the lack of detailed life cycle inventory (LCI) data and limited access to commercial life cycle assessment (LCA) tools. This work presents a predictive framework for estimating the environmental impacts of chemicals and processes during the early design stages when such information is unavailable. The framework combines Machine Learning (ML) models, Artificial Neural Networks (ANN), and eXtreme Gradient Boosting (XGBoost), trained on molecular descriptors and thermodynamic properties to predict four cradle-to-gate (production phase) endpoint metrics: Human Health Impact (HHI), Ecosystem Quality Impact (EQI), Global Warming Potential (GWP), and Resource Utilization Impact (RUI). To extend the analysis to the gate-to-gate (use phase) and gate-to-grave (end-of-life) phases, a power law regression model was developed to estimate the GWP scaling exponents for technologies as a function of the capacity/processing rate and energy consumption. Feature importance analysis using SHapley Additive exPlanations (SHAP) indicates that both molecular and thermodynamic descriptors contribute significantly to the prediction of environmental impacts. Two case studies were selected to demonstrate and validate the applicability of this framework. Overall, the results demonstrate the combined approach provides a practical tool for early-stage environmental screening of chemicals and processes.

**Graphical abstract:**

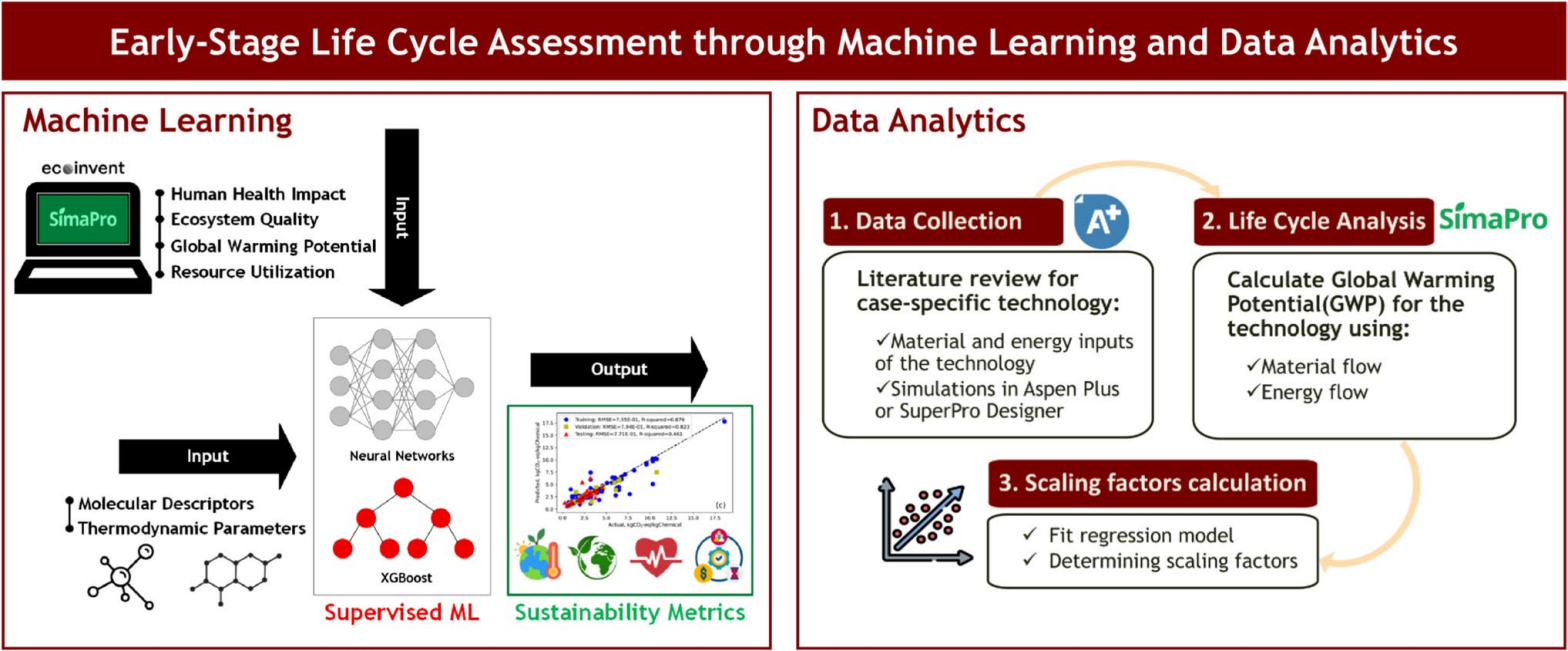

**Supplementary Information:**

The online version contains supplementary material available at 10.1007/s10098-026-03479-8.

## Introduction

The continued expansion of industrial activity worldwide has increased the demand for chemical products while also exacerbating the associated environmental burden during its production and use (Levi and Cullen 2018). Since 1990, global greenhouse gas emissions have increased substantially, reaching approximately 53 gigatons of CO_2_-equivalent in 2023 (Rivera et al. 2023). Industry accounts for roughly one-quarter of global greenhouse gas emissions (Lopez et al. 2023) and within the industry, the chemical sector is the largest energy consumer and a major source of direct CO_2_ emissions (“Chemicals,” IEA. Accessed 2026). Beyond climate impacts, chemical production and use can also release hazardous substances that contribute to environmental degradation and pose risks to ecosystems and human health through persistence and bioaccumulation. With the global chemical industry projected to double by 2050 relative to that of 2017, accelerating the transition toward more sustainable practices is extremely important (Zhang et al. 2024).

In response to these environmental challenges, various metrics have been developed to quantify and assess the environmental impacts associated with chemicals and industrial processes. The E-factor, proposed by Sheldon, relates the mass of waste generated to the mass of the product produced (Tobiszewski et al. 2015),whereas Process Mass Intensity (PMI) captures the total mass of materials used per unit mass of the product (Jimenez-Gonzalez et al. 2011). In addition to these mass-based metrics, the Atom Economy introduced by Trost quantifies the theoretical fraction of the reactant mass that is incorporated into the desired product (“The Atom Economy—A Search for Synthetic Efficiency”. 2025). While useful for rapid comparisons, these metrics are largely limited to the synthesis step and do not capture upstream burdens associated with producing inputs or downstream impacts during use and end-of-life phases. Life cycle assessment/analysis (LCA), as standardized in ISO 14040/14044, addresses these limitations by quantifying environmental impacts across defined system boundaries for chemicals and processes (Aboagye et al. 2025). It offers a comprehensive framework that spans the entire life cycle of a product, from raw material extraction to production (cradle-to-gate), through use (gate-to-gate), to end-of-life phase, whether managed by disposal (gate-to-grave) (Jolliet et al. 2003) or recovery and recycling (gate-to-cradle) (Wernet et al. 2009).

To date, extensive LCA studies have been conducted on hundreds of widely used chemicals, and well-established databases such as Ecoinvent compile cradle-to-gate inventories for nearly 2,000 chemicals across various chemical categories. However, a significant data gap remains for emerging chemicals or those still in the laboratory scale synthesis phase. In response, several predictive tools based on machine learning (ML) algorithms, such as neural networks (NNs) and decision trees, have been developed to estimate life cycle impacts directly from chemical properties(Sun et al. 2023). These ML-based methods represent a paradigm shift from traditional, process-based LCA(Calvo-Serrano et al. 2018a). Whereas conventional LCA constructs detailed inventories of material and energy flows and associated emissions by modeling each stage of the supply chain, from raw material extraction to end-of-life, ML-driven tools adopt a data-centric approach, learning correlations between chemical properties and environmental outcomes(Song et al. 2017).

In prior studies, Wernet et al. 2009, developed molecular structure-based models (MSMs) using multi-layer perceptron neural networks that predict cradle-to-gate indicators such as cumulative energy demand (CED) and global warming potential (GWP) directly from simple molecular descriptors, providing route-independent impact profiles based solely on chemical structure. Calvo-Serrano et al. 2018a and b, enhanced this approach by integrating mixed-integer programming frameworks that combine molecular descriptors with thermodynamic properties, improving prediction accuracy for life cycle assessment (LCA) and facilitating integration into process design tools(Calvo-Serrano et al. 2018b). Extending beyond petrochemicals, P. Karka et al. 2022 applied decision trees and artificial neural networks to biomass and biorefinery-based processes, incorporating biomass type, product information, and process chain descriptors to extend impact predictions beyond molecular structure alone(Karka et al. 2022). Kleinekorte et al. 2023 introduced a hybrid model combining an encoder–decoder neural network pretrained on large structural databases with Gaussian process regression, allowing process-specific predictions of global warming impact that distinguish between production routes for the same chemical, addressing limitations of earlier MSMs (Kleinekorte et al. 2023). Most recently, Gao et al. 2026 demonstrated that graph neural networks (GNNs) operating directly on molecular graphs outperform traditional Quantitative Structure Property Relationship models in predicting multiple life cycle impact categories, including climate change, and can predict up to fifteen impact categories simultaneously in a multi-task learning framework(Gao et al. 2026).

While these studies represent important advances, key limitations continue to hinder their application to early-stage design decisions. Most existing approaches focus exclusively on cradle-to-gate impact prediction, providing only production-stage environmental footprints rather than full life cycle profiles. This is a significant gap given that use-phase and end-of-life contributions can dominate the total impact of many chemical processes. Furthermore, the predominant reliance on molecular structure descriptors means that thermodynamic properties such as boiling point, heat capacity, and enthalpy-related parameters are frequently overlooked, despite their direct relevance to separation difficulty, energy demand, and material flows in chemical manufacturing. These properties are not merely supplementary. They encode the physical behavior that determines how much energy a process consumes and omitting them limits the physical interpretability and predictive scope of the resulting models. In cases where both molecular and thermodynamic descriptors are considered, models often require large feature sets that are impractical for early-stage applications, where many inputs are unavailable, uncertain, or simply impossible to measure for chemicals that have not yet been synthesized. This tension between model richness and practical accessibility represents a fundamental barrier to the adoption of ML-based LCA tools in real design workflows.

This work addresses these limitations through two principal contributions. First, a data-driven framework is developed that extends predictive LCA beyond the cradle-to-gate stage by introducing a methodology to estimate environmental impacts at the gate-to-gate and gate-to-grave phase, enabling a more complete life cycle scope. Second, for the cradle-to-gate stage, the proposed model is built on a compact set of physically meaningful features that combine molecular structure descriptors with key thermodynamic properties, making the approach both simpler and more practical than existing feature-rich alternatives. Because the selected descriptors are readily accessible from standard chemical databases and estimation tools, the framework is applicable not only to novel chemicals still in the design phase but also to existing chemicals in cases where access to commercial LCA is unavailable or cost prohibitive. The following sections describe the overall workflow, the data collection and model development procedures for both the cradle-to-gate phase and the gate-to-gate phase, the results and their interpretation, and two case studies that demonstrate and validate the application of the developed framework.

## Methodology

This section presents the computational framework developed to predict the environmental impacts of chemicals across their full life cycle. The framework is structured around three sequential phases: (1) A production phase, in which ML models are trained to predict cradle-to-gate environmental impact metrics from molecular and thermodynamic descriptors; (2) a use and end-of-life phase, in which a power law regression model is used to estimate the GWP associated with technologies; and (3) a validation phase, in which the complete framework is applied to two case studies to assess its predictive accuracy. Each phase is described in detail in the following subsections.

### Production phase

**Fig. 1:**
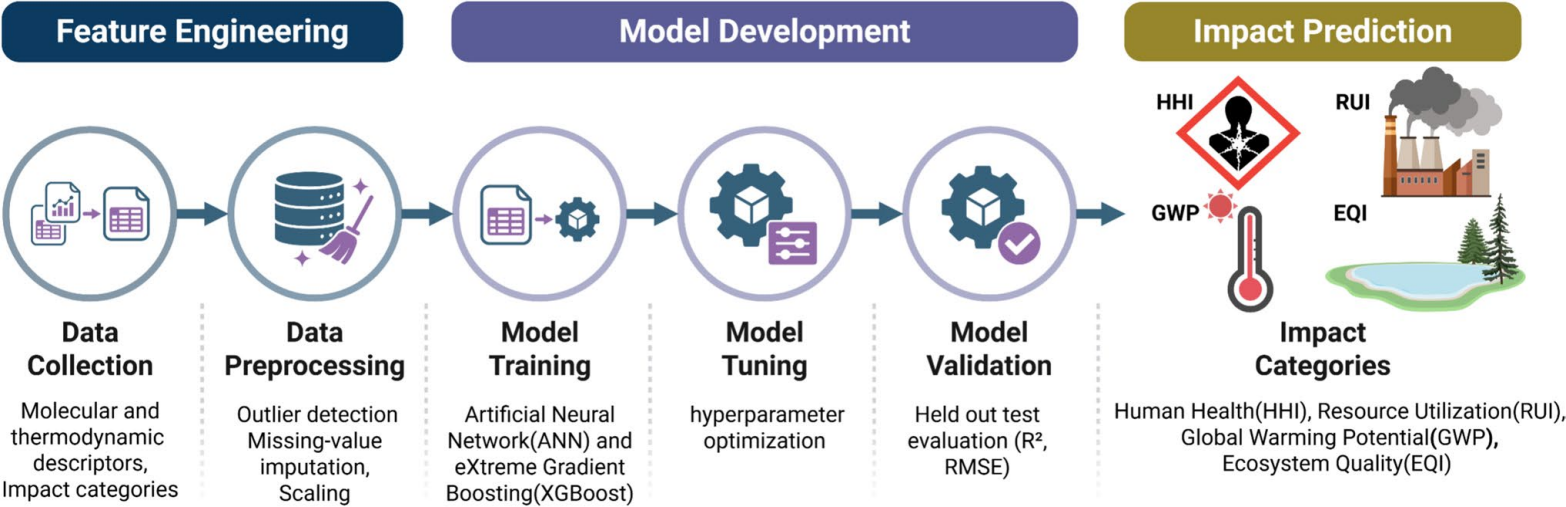
Machine learning framework for production phase environmental impact assessment

This phase covers the environmental impacts from raw material extraction up to the point at which the materials reach the facility gate for use in product manufacturing, encompassing the cradle-to-gate scope of the life cycle. Fig. 1 presents an overview of the production phase framework, and the methodology is described in the following subsections.

### Data collection for production phase

A dataset of 500 commonly used organic solvents spanning multiple chemical families, including alcohols, esters, hydrocarbons, and ethers were compiled. This chemical diversity was deliberately incorporated to ensure that the trained models learn structure–property relationships that generalize across functional group classes rather than being confined to a single chemical family. For each compound, data collection was carried out in two steps: extraction of the feature set and extraction of the label set. For the feature set, the SMILES string and molecular formula were first retrieved using CIRpy, a Python library that interfaces with the Chemical Identifier Resolver (CIR). The retrieved SMILES strings were then used to compute 23 thermodynamic properties, including critical temperature, critical pressure, critical volume, heat capacity, boiling point, and standard Gibbs free energy, using the Python libraries chemicals (v1.14) and thermo (v0.2.26), which draw from well-established databanks including the National Institute of Standards and Technology (NIST), the Design Institute for Physical Properties (DIPPR), and PubChem, providing access to thermodynamic data for over 20,000 compounds. An additional 200 molecular descriptors were computed using RDKit (v2023.03.3), an open-source cheminformatics toolkit widely used in drug discovery and toxicological studies, capturing structural and physicochemical information such as molecular weight, partial charge distributions, functional group counts, number of rotatable bonds, and topological indices.

For the label set, four endpoint environmental impact metrics were extracted for each chemical: Human Health Impact (HHI), Ecosystem Quality Impact (EQI), Global Warming Potential (GWP), and Resource Utilization Impact (RUI). These metrics were obtained at cradle-to-gate scope using the IMPACT 2002+ life cycle assessment methodology in SimaPro®, a commercial LCA software package, with the Ecoinvent database serving as the background life cycle inventory system.

### Data processing

Prior to model development, the extracted dataset was subjected to a structured preprocessing pipeline to address missing values, outliers, and feature redundancy. Missing values were estimated using a k-means clustering algorithm that partitioned the dataset into six clusters based on molecular weight. The optimal number of clusters was determined by comparing within-cluster imputed values against known values from the DIPPR database, selecting the configuration that minimized imputation error. Within each cluster, missing entries were replaced with the cluster average for that feature. This approach produces more chemically representative estimates than global imputation, as it draws only from compounds with similar molecular weight and therefore comparable physicochemical behavior. Following imputation, outliers were identified and removed using z-score normalization. Since the environmental impact metrics do not follow a normal distribution, a log-transformation was first applied to the label data before computing z-scores. Data points exceeding three standard deviations from the mean were removed, yielding a final dataset of 350 chemicals for model development.

The feature set was then scaled to a uniform range to prevent high-magnitude features from disproportionately influencing model training. To reduce the initial feature space, principal component analysis was first used to determine the number of components needed to retain 90% of the data variability, which indicated that nine components were sufficient. Based on this, five features were selected from each feature type, thermodynamic properties and molecular descriptors, giving a total of ten features per model. Sequential backward feature selection with linear regression and mean squared error as the scoring criterion was then applied independently for each impact metric, ensuring that the retained features reflect the specific physicochemical drivers of HHI, EQI, GWP, and RUI, respectively. The full mathematical formulation and implementation details are provided in Supplementary Information Section S.1.

### Model development

The dataset was partitioned into training (80%), validation (10%), and test (10%) subsets. The training set was used to fit the models, the validation set guided hyperparameter optimization, and the held-out test set provided an independent evaluation of generalization performance. Two ML algorithms were employed to predict the four endpoint impact metrics, one using an Artificial Neural Network (ANN) and one using eXtreme Gradient Boosting (XGBoost). The ANN works by passing data forward through a series of layers, where each node takes in outputs from all nodes in the previous layer, combines them through a weighted sum, and passes the result through an activation function(Chen et al. 2025). During training, the model compares its predictions against the actual values and works backwards through the network, a process called backpropagation, adjusting the weights layer by layer to progressively reduce the prediction error. XGBoost, on the other hand, works by building an ensemble of decision trees sequentially, where each new tree is trained to correct the errors made by the previous ones(Wiens et al. 2025). Unlike the ANN, which refines a single continuous model through weight updates, XGBoost improves its predictions by adding new trees that target the remaining residual errors, with a regularization term in the objective function that penalizes complexity and prevents overfitting as the ensemble grows.

For both models, hyperparameter optimization was carried out using the hyperopt library (v0.2.7), which employs Bayesian optimization to efficiently search the hyperparameter space with validation set mean squared error as the objective function. For the ANN, implemented in TensorFlow with the Adam optimizer, the tuned hyperparameters included the learning rate, number of hidden layers, number of neurons per layer, dropout rate, activation function, loss function, batch size, and number of epochs. The optimal hyperparameter configurations identified for each impact metric are reported in Supplementary Information Section S.2, Table S1. For XGBoost, the tuned hyperparameters included maximum tree depth, learning rate, number of estimators, minimum child weight, and subsample fraction. To quantify predictive uncertainty, a bootstrap resampling approach was employed with 1000 iterations, randomly sampling 50% of the dataset in each iteration. A 95% confidence interval was estimated providing an empirical measure of predictive variability that allows direct comparison between model predictions and the actual data distribution.

Once the model was trained and hyperparameters optimized, its performance was evaluated using coefficient of determination (R^2^) and the Root Mean Squared Error (RMSE). The R^2^ provided an indication of the proportion of variance explained by the model, while RMSE quantified the average prediction error, offering a concrete measure of model performance. To better understand how this model uses the selected features, we additionally analyzed feature importance using SHapley Additive exPlanations (SHAP). This SHAP determines the importance of each feature by quantifying how much it influences the model’s output. It examines the difference in the model’s prediction when a feature is present versus when it is left out(Lundberg and Lee 2017).

### Use and end-of-life phases

**Fig. 2:**
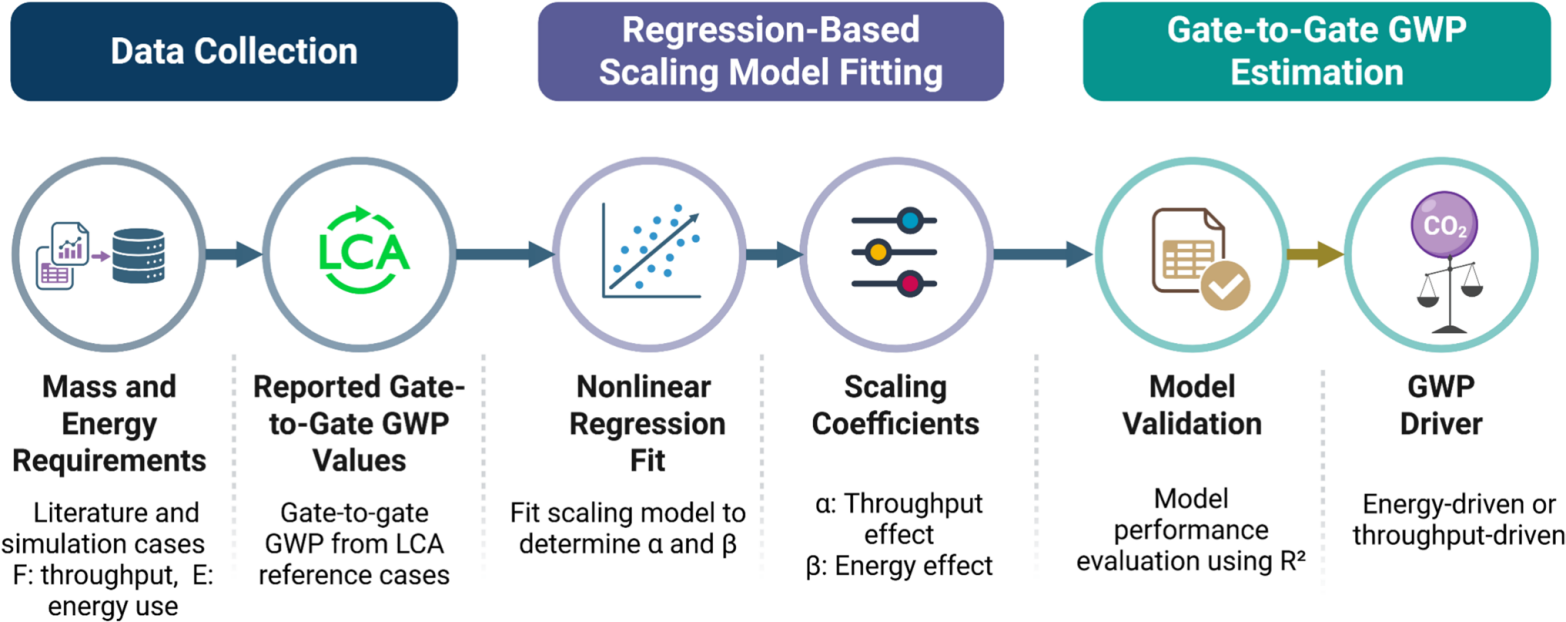
Data analytical framework for use phase and end-of-life phase environmental impact assessment

This phase evaluates the environmental impacts associated with the use and end-of-life stages of a chemical’s life cycle. The primary drivers of environmental impact in this phase are the technologies involved in the process, particularly the quantity of raw materials consumed, and the energy demanded during operation. The degree to which each of these factors influence GWP varies across technologies, and understanding this sensitivity forms the basis for a developing power law scaling model to estimate gate-to-gate GWP as a function of readily available process operating parameters. Figure 2 presents an overview of this phase, and the methodology is described in the following subsections.

### Data collection for the technologies

Two categories of data were collected for this phase: The operational parameters of each technology, specifically process throughput (F) and energy consumption (E), and the corresponding gate-to-gate GWP values obtained from LCA reference cases. Together, these form the input–output pairs needed to fit the scaling model. For the process throughput (F) and energy consumption (E), these data were sourced from a comprehensive review of peer-reviewed literature, industrial reports, and published case studies reporting mass and energy balances for a range of technologies, including distillation, pervaporation, drying, and membrane filtration. Since the power law scaling model employed in this work is analogous to classical cost estimation correlations in chemical engineering (Chaouki and Sotudeh-Gharebagh 2021), the throughput parameter F was defined following the same convention used in cost scaling. That is, the parameter that most meaningfully represents the size or throughput of the equipment. It is important to note that F represents the total materials processing rate or capacity of the technology and E represents the total energy consumed in that technology. For cases where sufficient mass and energy balance data were not available in the literature, the process was simulated using Aspen Plus or SuperPro Designer to obtain the required values. For the gate-to-gate GWP values, the same literature sources were first consulted. Where GWP values were not explicitly reported, they were independently calculated using SimaPro with the Ecoinvent database as the background inventory system.

To expedite the literature search, generative AI tools including ChatGPT, Consensus, Perplexity, and DeepSeek were used solely to identify potentially relevant papers. These tools were not used to generate or extract any data. For every paper suggested by these tools, the full publication was independently located, downloaded, and read to confirm that it was a real, published article and that the content was relevant before any data were extracted.

### Regression-based model

Following data collection, a power law scaling relationship was developed to relate the gate-to-gate GWP of technology to its process throughput and energy requirements. Drawing on the analogy with classical cost estimation correlations in chemical engineering, where equipment cost scales with capacity through a power law exponent, the GWP of a technology is expressed in Eq. 1 as:

1 $$\left( {\frac{{GWP_{new} }}{{GWP_{ref} }}} \right) = \left( {\frac{{F_{new} }}{{F_{ref} }}} \right)^{\alpha } \left( {\frac{{E_{new} }}{{E_{ref} }}} \right)^{\beta }$$

Where F denotes the process throughput, E denotes energy consumption, and the subscripts *new* and *ref* refer to the case being estimated and the selected reference case, respectively. The exponents α and β are the scaling coefficients that capture the sensitivity of GWP to changes in throughput and energy consumption, respectively. To determine α and β, equation 1 was linearized by taking the natural logarithm of both sides, transforming the power law relationship into a linear form:

2 $$\ln Y = \alpha \ln X_{1} + \beta lnX_{2}$$

where $$X_{1} = \frac{{{\mathrm{F}}_{{{\mathrm{new}}}} }}{{{\mathrm{F}}_{{{\mathrm{ref}}}} }}$$, $$X_{2} = \frac{{{\mathrm{E}}_{{{\mathrm{new}}}} }}{{{\mathrm{E}}_{{{\mathrm{ref}}}} }},$$ and $$Y = \frac{{{\mathrm{GWP}}_{{{\mathrm{new}}}} }}{{{\mathrm{GWP}}_{{{\mathrm{ref}}}} }}$$

Prior to regression, all data points were normalized against the data point with the highest material processing rate in the dataset. The model performance was assessed using the R^2^, and the resulting values of α and β identify whether the GWP of a given technology is primarily driven by changes in process throughput or energy consumption.

### Process description of case studies

To demonstrate and validate the proposed framework, two case studies are presented. The first case study focuses on the recovery of isopropanol from a pharmaceutical waste stream, using the superstructure-based solvent recovery framework developed by Chea et al.(Chea et al. 2020). From the set of possible recovery pathways identified in that work, the distillation–pervaporation route was selected for analysis. The second case study involves the separation of an ethanol–water mixture using hybrid distillation–pervaporation configurations, based on the life cycle assessment study of Do Thi and Tóth (Do Thi and Toth 2024).

#### Case study 1: Isopropanol (IPA) recovery from a pharmaceutical waste stream

**Fig. 3:**
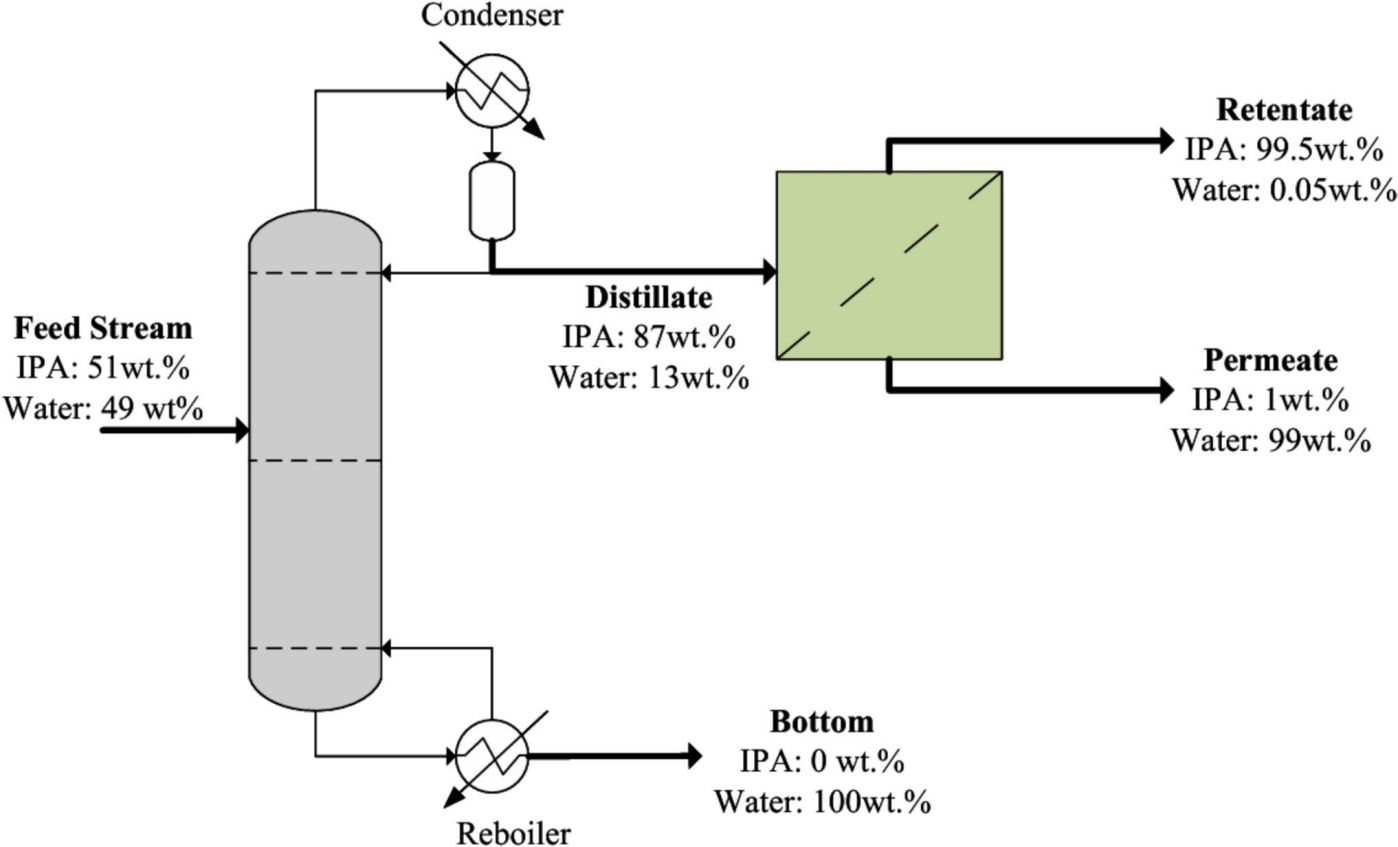
Process flow diagram for the distillation–pervaporation pathway for IPA recovery from a pharmaceutical waste stream

In this case study, a pharmaceutical waste stream generated from the celecoxib synthesis process is considered, consisting of an isopropanol–water mixture in which isopropanol (IPA) accounts for 51 wt.% at a total feed rate of 1000 kg/hr. The waste stream originates from downstream purification steps in the celecoxib manufacturing process, where organic solvents are extensively used during synthesis, washing, and drying operations. The recovery of isopropanol from this waste stream is particularly important because solvents represent a significant portion of the process mass and are often discarded after a single use. To recover IPA from this waste stream, a superstructure consisting of multiple candidate separation technologies was proposed. The distillation–pervaporation pathway identified within this superstructure was selected for analysis in the present study, as illustrated in Fig. 3.

#### Case Study 2: Ethanol–water hybrid separation systems

**Fig. 4:**
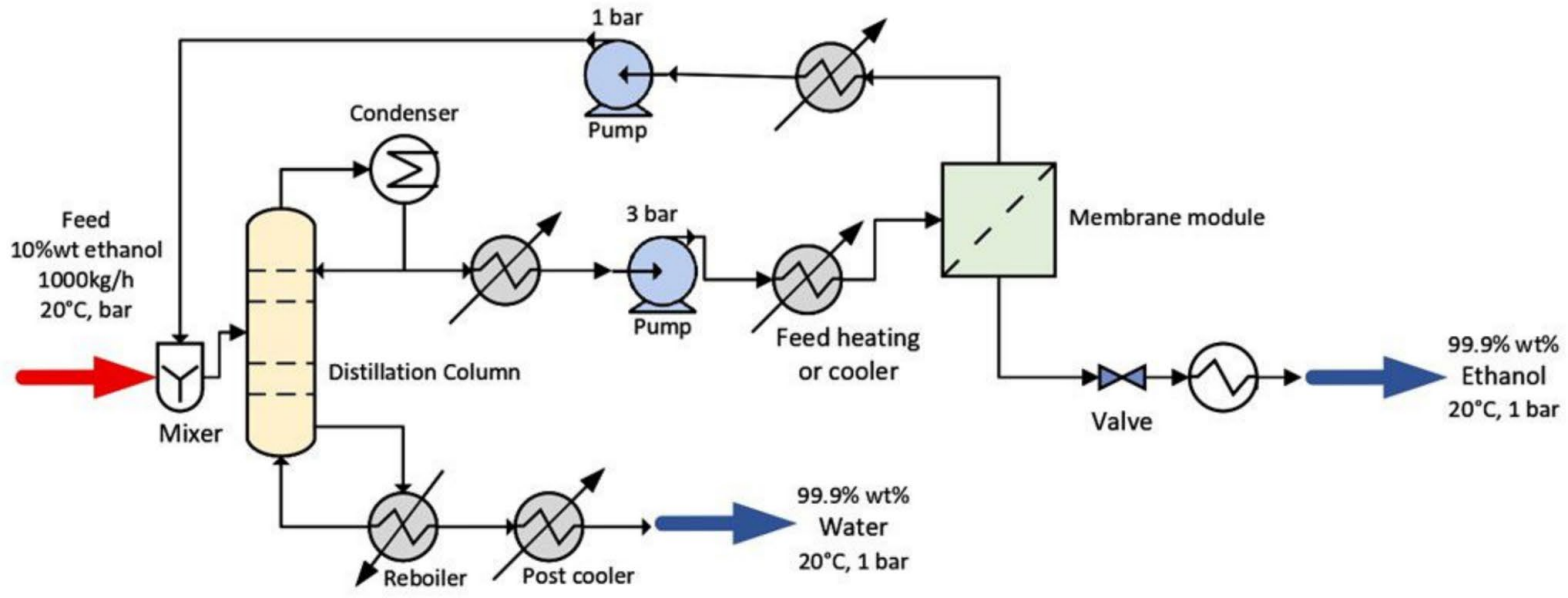
Process flow diagram of D+PV system

This case study examines the separation of a 10 wt.% ethanol–water mixture at a feed rate of 1000 kg/h, at 20°C and 1 bar, with a target product purity of 99.9 wt.% for both ethanol and water. Three hybrid configurations are evaluated: distillation coupled with pervaporation (D+PV), distillation followed by pervaporation and a second distillation column (D+PV+D), and the same configuration with partial heat integration (D+PV+D+HI). In the first configuration, D+PV, the feed enters a 40-stage distillation column optimized using the UNIQUAC thermodynamic model, producing 99.9 wt.% water as the bottom product. The distillate, enriched in ethanol but limited by the azeotropic point to approximately 87.2 wt.%, is fed into a hydrophilic pervaporation membrane system consisting of multiple cross-flow modules in series, each with a membrane surface area not exceeding 10 m^2^, and operated at 90 °C and 3 bar on the feed side with a permeate pressure of 0.008 bar, producing 99.9 wt.% ethanol as the retentate. Figure 4 presents the process flow diagram for this configuration.

**Fig. 5:**
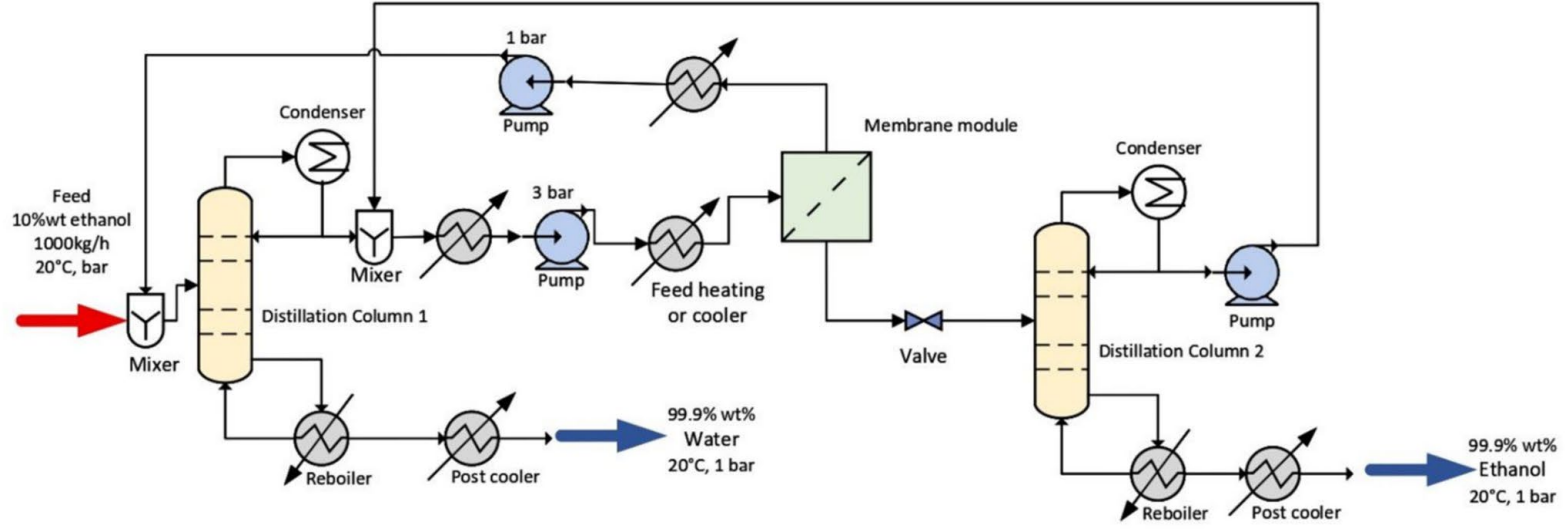
Process flow diagram of D+PV+D system

**Fig. 6:**
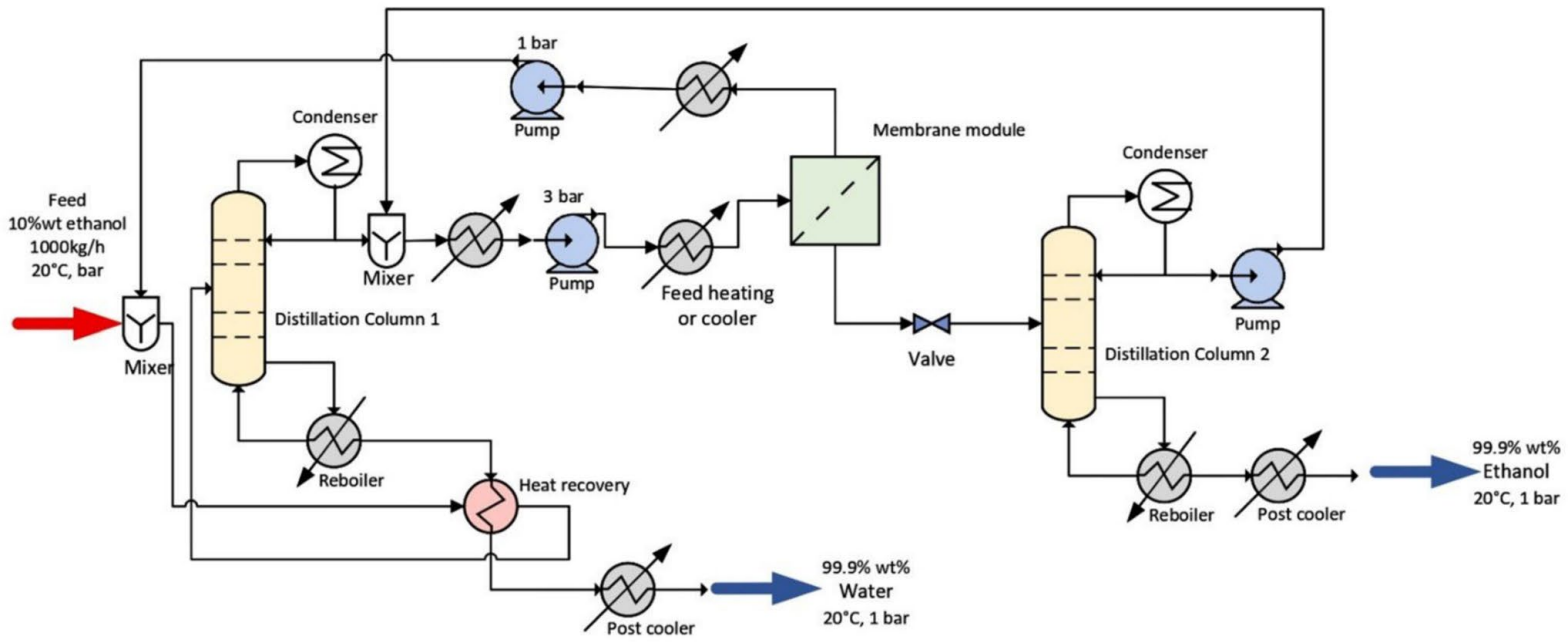
Process flow diagram of D+PV +D+HI system

In the second configuration, D+PV+D, the pervaporation step moves the composition through the azeotropic point, and the retentate is fed into a second distillation column to achieve the final product purity, requiring a total PV membrane area of only 130 m^2^ compared to 200 m^2^ for the D+PV system. Figure 5 presents the process flow diagram for this configuration. In the third configuration, D+PV+D+HI, partial heat integration is introduced by recovering thermal energy from the bottom product of the first distillation column to preheat the incoming feed, reducing total heat requirements by 16.5% compared to the D+PV+D system without heat integration. Figure 6 presents the process flow diagram for this configuration. Extensive details of the process simulation for each configuration are available in the original study by Do Thi and Tóth. The mass and energy balance data reported in their study are used as inputs to our framework to estimate the GWP of the ethanol–water separation process across all three configurations, and the predicted values are compared against the SimaPro calculated GWP values from the original study to assess the predictive accuracy of the framework.

## Results and discussion

The framework developed in this work provides an early-stage environmental impact estimation approach for situations where detailed life cycle inventory data and commercial LCA tools are not available. Rather than reproducing exact LCA outputs, the objective is to provide reliable directional estimates that support sustainability informed decision-making during the earliest stages of chemical and process design, particularly for emerging chemicals for which detailed inventories are unavailable.

Results are first presented for the molecular descriptors and thermodynamic properties selected for each impact metric, followed by the predictive performance of the ANN and XGBoost models for the cradle-to-gate phase and SHAP analysis of feature importance. The gate-to-gate power law scaling model results for the technologies are then discussed, followed by two case studies validating the complete framework against published life cycle assessment results.

### Cradle-to-gate model results

Table 1 summarizes the thermodynamic properties and molecular descriptors selected through sequential backward feature selection for each impact metric. Five thermodynamic properties and five molecular descriptors were retained for each model, resulting in ten input features per impact category.

**Table 1:** Selected features for each label metric using a sequential backward feature selection approach

Metric	Selected features	
	*Thermodynamic properties*	*Molecular descriptors*
HHI	heat of vaporization, heat capacity, partition coefficient, acentric factor, critical temperature	Chi0n, HallKierAlpha, SMR_VSA7, VSA_Estate6, NumValenceElectrons
EQI	heat capacity, standard formation enthalpy (gas), boiling Point, critical temperature, critical volume	Chi2v, BertzCT, HallKierAlpha, qed, fr_halogen
GWP	Heat capacity, boiling point, partition coefficient, critical temperature, critical molar volume	BertzCT, ExactMolWt, HallKierAlpha, PEOE_VSA6, NOCount
RUI	heat capacity, boiling point, partition coefficient, critical pressure, critical temperature	ExactMolWt, MaxAbsPartialCharge, MaxPartialCharge, SMR_VSA2, NumRotatableBonds,

Figure 7 shows the parity plots for each impact metric from the ANN model. The model performs best for HHI (Fig. 7a), achieving a test R^2^ of 0.957 with RMSE values similar across the training, validation, and test sets, indicating consistent and reliable generalization to unseen chemicals. The 95% confidence interval for HHI predictions ranges from 0.606 to 12.138 × 10^−6^ DALY (Disability-adjusted lifetime years)/kgchem, closely tracking the actual data range of 0.545 to 12.251 × 10^−6^ DALY/kgchem. For EQI (Fig. 7b) although the RMSE values remain within acceptable limits, suggesting general reliability, the model exhibits a significant discrepancy in the R^2^ value for the training, validation, and testing sets. This variance indicates a need for refinement in the model to achieve a more dependable R^2^ value for EQI predictions. The predictions for EQI range from 0.022 to 3.0 PDF (Potentially Disappearing Fractions).m^2^. yr/kgchem. The GWP model shows moderate predictive performance with a test R^2^ of 0.599 and an RMSE of 1.260 (Fig. 7c). Although the model overfits the training data, as reflected in the gap between training and test R^2^ values, it achieves closer agreement with true values than the EQI model, with predictions ranging from 0.816 to 8.375 kg CO_2_-eq/kgchem at the 95% confidence level. For RUI (Fig. 7d), which can also be interpreted as the Cumulative Energy Demand for chemical production, the model performs well with predictions ranging from 36.67 to 169.60 MJ-primary/kgchem, closely aligned with the actual data range of 34.867 to 175.910 MJ-primary/kgchem.

**Fig. 7:**
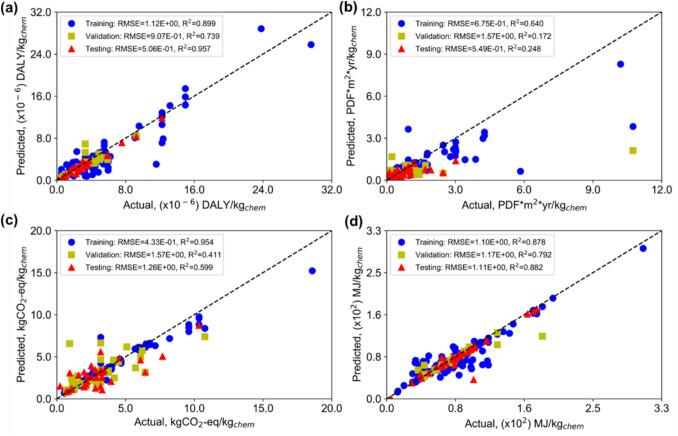
Parity plot for each metric from the ANN model. **a** HHI, **b** EQI, **c** GWP, **d** RUI

**Fig. 8:**
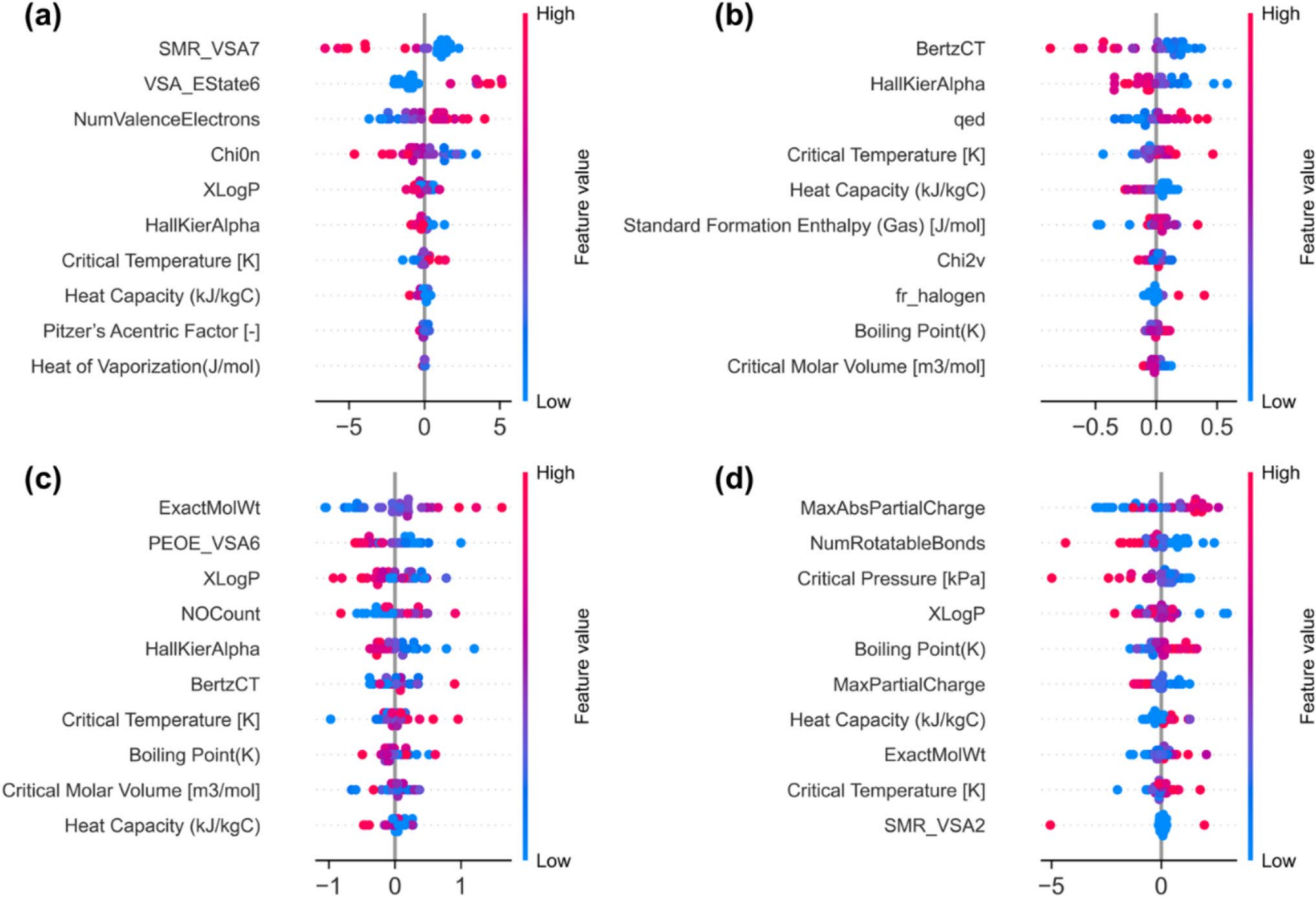
Feature importance for ANN model using the SHAP analysis **a** HHI **b** EQI **c** GWP **d** RUI

In Fig. 8 the SHAP-based feature importance for the ANN model across all four environmental impact metrics is shown. Each point corresponds to one sample for a given feature, where the horizontal position indicates the strength and direction of the feature’s influence on the prediction, and the point color indicates the feature value, blue for low values and red for high values. Positive contributions appear to the right of the baseline and negative ones to the left.

For HHI (Fig. 8a), the most influential features are the molecular descriptors SMR_VSA7, NumValenceElectrons, and VSA_EState6. SMR_VSA7 represents the van der Waals surface area weighted by molar refractivity and therefore reflects molecular size and polarizability, which influence intermolecular interactions and partitioning behavior. NumValenceElectrons represents the total number of valence electrons in the molecule and is associated with overall molecular composition and bonding structure. VSA_EState6 combines electrotopological state indices with accessible surface area, capturing how electronic character is distributed across the molecular surface. Together, these descriptors characterize structural and electronic properties that influence how chemicals partition among environmental compartments such as air, water, soil, and biological media, their interactions with organic matter, and their potential to bioaccumulate. In life cycle impact assessment, human health damage factors are determined by the combined effects of environmental fate, exposure pathways, and toxicity, which depend strongly on these underlying molecular properties. For EQI (Fig.8b), BertzCT, HallKierAlpha, and qed are the most influential features. BertzCT is a topological descriptor commonly interpreted as a measure of molecular complexity, while HallKierAlpha is a connectivity-related descriptor reflecting aspects of molecular topology and atom-type contributions. The qed descriptor summarizes several molecular properties, including molecular weight, lipophilicity, polar surface area, hydrogen-bonding terms, rotatable bonds, aromaticity, and structural alerts. Together, these descriptors are associated with molecular properties that influence environmental distribution, persistence, and ecological exposure, all of which are relevant to ecosystem quality impacts in LCIA. For GWP (Fig. 8c) and RUI (Fig. 8d), both molecular and thermodynamic descriptors contribute substantially to model predictions. Descriptors associated with molecular size, polarity, charge distribution, and flexibility, together with boiling point, critical properties, and heat capacity, indicate that these two metrics are strongly influenced by physicochemical factors governing process energy demand and resource use.

**Fig. 9:**
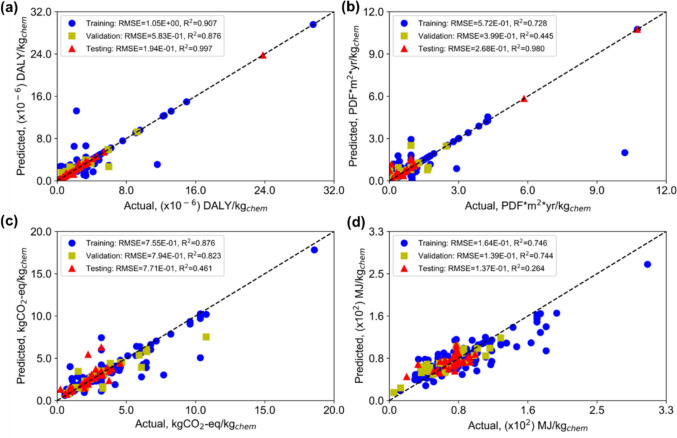
Parity plot for each metric from the XGBoost model. **a** HHI, **b** EQI, **c** GWP, **d** RUI

For the XGBoost model, as seen in Fig. 9, a similar trend is observed across the four impact metrics, with the model performing best for HHI (Fig. 9a), achieving a confidence interval of 0.632 to 12.374 × 10^−6^ DALY/kgchem that closely tracks the actual data range of 0.545 to 12.251 × 10^−6^ DALY/kgchem. Notably, the XGBoost model outperforms the ANN for EQI (Fig. 9b), with a tighter confidence interval of 0.022 to 3.010 PDF·m^2^·yr/kgchem and shows less overfitting for GWP (Fig. 9c), with predictions ranging from 0.810 to 9.048 kg CO_2_-eq/kgchem. For RUI however, the ANN outperforms XGBoost, with the XGBoost confidence interval of 44.934 to 153.296 MJ-primary/kgchem underestimating the upper range of the actual data compared to the ANN interval of 36.67 to 169.60 MJ-primary/kgchem (Fig. 9d). Overall, both models demonstrate their strongest predictive performance for HHI, and their complementary strengths across the remaining metrics suggest that neither algorithm is universally superior for environmental impact prediction.

**Fig. 10:**
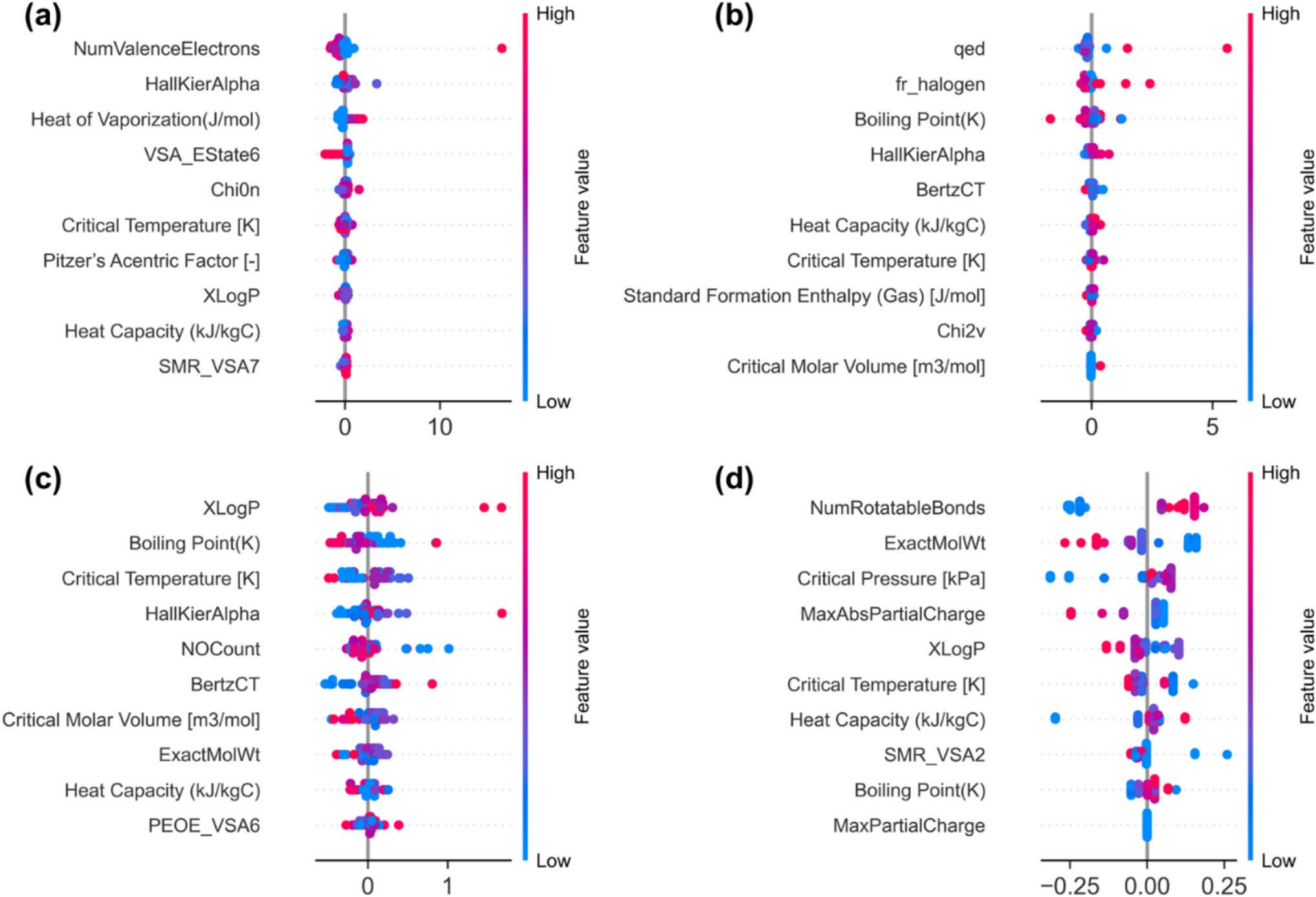
Feature importance for XGBoost model using the SHAP analysis **a** HHI **b** EQI **c** GWP **d** RUI

For the XGBoost SHAP analysis shown in Fig. 10, HHI (Fig. 10a), shows the most influential features as NumValenceElectrons and HallKierAlpha, followed by heat of vaporization and VSA_EState6. The narrow spread of most SHAP values suggests that many variables contribute modestly, while a few samples show stronger positive contributions at higher feature values. These descriptors capture electronic structure, molecular topology, and intermolecular interaction strength, which are properties associated with chemical reactivity and toxicity-related mechanisms that influence human health impact indicators. For EQI (Fig. 10b), qed appears as the dominant feature with the widest SHAP spread, indicating a strong influence on the predicted values. Additional contributions arise from fr_halogen, boiling point, and HallKierAlpha, suggesting that both structural descriptors and thermophysical properties contribute to the prediction of ecological impacts. For GWP (Fig. 10c), XLogP shows the largest SHAP magnitude and therefore the strongest influence on the model output. Other important predictors include boiling point and critical temperature, indicating that properties associated with volatility and thermodynamic behavior influence the predicted global warming impacts. The clustering of points around zero for several other variables suggests a smaller marginal contribution relative to the dominant descriptors. For RUI (Fig. 10d), the most influential features include NumRotatableBonds, ExactMolWt, and critical pressure. These variables exhibit a broader SHAP distribution compared with other features, indicating stronger contributions to model predictions. These descriptors reflect molecular size, flexibility, and thermodynamic behavior, which are associated with resource and energy requirements during chemical production.

### Gate-to-gate model results

The power law scaling model defined by Eq. 1 was applied to operational data for several technologies, with the scaling coefficients α and β determined independently for each technology through linear regression of the linearized form given in Eq. 2. The coefficient α captures the sensitivity of GWP to changes in the process rate F, and β captures the sensitivity of GWP to changes in energy consumption E. Together with the R^2^ value of the regression, these coefficients characterize the dominant drivers of environmental impact for each technology. The resulting coefficients and R^2^ values are summarized in Table 2.

**Table 2:** Scaling coefficients α and β and coefficients of determination R^2^ obtained from regression of Eq. 1 for each technology

Technology	α (Throughput sensitivity)	ß (Energy sensitivity)	R^2^ (coefficients of determination)
Distillation	0	0.702	0.9442
Pervaporation	0.0154	0.4114	0.894
Dryer	0.074	0.489	0.7911
Membrane	0.9102	1.101	0.9883

Tables 3 and 4 present the operational data compiled from published LCA and process simulation studies for distillation and pervaporation, respectively. Although the number of data points for each technology is limited, with 9 data points for distillation 5 data points for pervaporation, only pilot and industrial-scale cases with verified mass and energy balances were retained, since laboratory-scale systems may exhibit energy consumption patterns that are not representative of commercial operation. Expanding these datasets in future work would improve the statistical robustness and generalizability of the scaling models.

**Table 3:** Data points for distillation obtained from literature

	Feed Rate (kg/s)	Energy (kW)	GWP (kg CO_2_-eq/kgchem)
Chea et al. (Chea et al. 2020)	0.2778	1175.3	0.2723
Do Thi and Toth. (Do Thi and Toth 2024)	0.2778	223.3	0.0706
Caballero-Sanchez et al. (Caballero-Sanchez et al. 2024)	0.0130	20.1	0.1295
Cavanagh et al. (Cavanagh et al. 2014)	0.1261	407.2	0.2067
Cavanagh et al. (Cavanagh et al. 2014)	0.4856	324.7	0.0500
Cavanagh et al. (Cavanagh et al. 2014)	0.3089	1022.9	0.2219
Arenas-Grimaldo et al.(Arenas-Grimaldo et al. 2024)	0.21	2263	1.94
Arenas-Grimaldo et al.(Arenas-Grimaldo et al. 2024)	1.24	5475	0.793
Arenas-Grimaldo et al.(Arenas-Grimaldo et al. 2024)	1.45	51360	6.35

**Table 4:** Data points for pervaporation obtained from literature

	Flux (L/m^2^.h)	Energy (kJ/h)	GWP (kg CO_2_-eq/kgchem)
Meng et al.(Meng et al. 2020)	4.3357	1.22E+07	1.46E-02
Lee et al.(Lee et al. 2016)	1.3870	2.05E+05	5.500E-03
Norkobilov et al.(Norkobilov et al. 2017)	0.262	1.54E+06	7.420E-03
Norkobilov et al.(Norkobilov et al. 2017)	0.2876	6.16E+05	3.160E-03
Do Thi and Toth(Do Thi and Toth 2024)	1.3000	1.66E+05	1.320E-03

For distillation, the fitted coefficients are α = 0 and β = 0.702 with R^2^ = 0.9442. Details of the regression procedure used to determine the fitted coefficients α and β are provided in the Supplementary Information. The positive value of β indicates that the predicted gate-to-gate GWP increases as energy consumption increases, which is consistent with the well-known thermal intensity of distillation, where reboiling and condensing duties dominate process energy requirements. In contrast, α converged to the imposed lower bound of zero, indicating that throughput did not emerge as a significant independent contributor within the fitted dataset once the effect of energy was accounted for. It is also important to note that this result is specific to the GWP metric considered here. For other environmental metrics, such as resource utilization or ecosystem quality impacts, the fitted coefficients may differ because the underlying drivers of those impacts are not necessarily the same as those governing GWP.

**Fig. 11:**
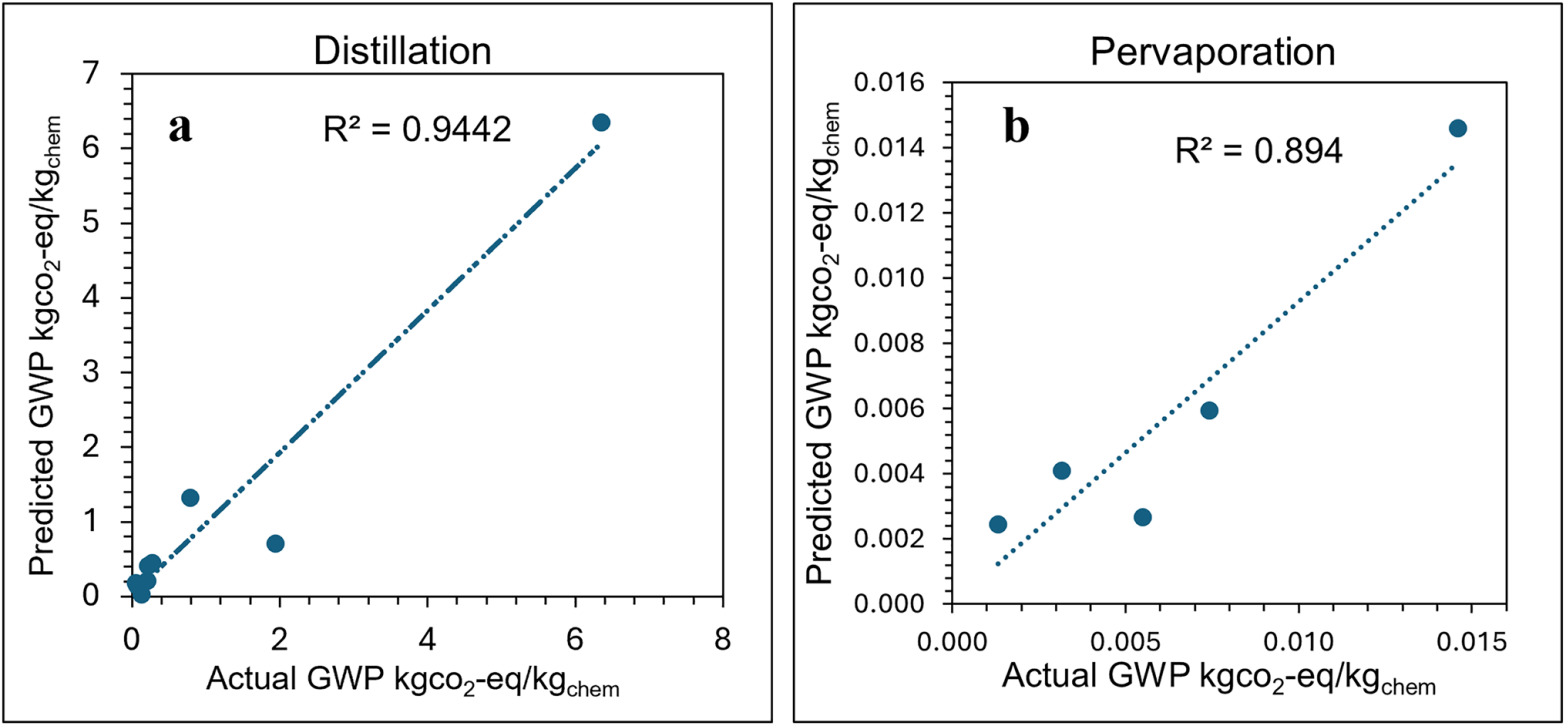
Regression plots **a** Distillation unit **b** Pervaporation unit

The regression plot (Fig. 11a) shows that the model reproduces the overall trend of the reference data well. The highest GWP case lies exactly on the line for the fitted relationship because it corresponds to the reference case used to normalize the scaling model. Greater scatter is observed among the lower GWP cases, including one mid-range case that is noticeably underpredicted, while the fitted relationship remains consistent with the overall trend in the dataset.

For pervaporation, the fitted coefficients are α = 0.0154 and β = 0.4114 with R^2^ = 0.894. The positive β indicates that GWP increases with energy consumption, consistent with the energy demands associated with feed heating and vacuum or sweep-gas operation in pervaporation systems. The small but nonzero α indicates that permeation flux contributes modestly to GWP, suggesting that membrane area and operating flux play a secondary but present role in determining the environmental impact of pervaporation. The regression plot in (Fig. 11b) shows a generally consistent trend across the fitted data range. The highest GWP case aligns exactly with the fitted relationship because it corresponds to the reference case used for normalization, while the remaining cases follow the same general trend with moderate deviation. Given the limited number of available reference points, the ${\mathrm{R}}^{2}$ value of 0.894 indicates that the scaling model provides a reasonable representation of the pervaporation GWP behavior across the fitted range.

### Case studies results

#### Case study 1: Isopropanol (IPA) recovery from a pharmaceutical waste stream

In this case study, we applied the proposed framework to estimate the GWP of the 1 kg of IPA recovered compare the predictions against SimaPro calculated values as a reference. Table 5 presents the predicted GWP from the framework alongside the SimaPro values for the cradle-to-gate, gate-to-gate, and total phases. Detailed calculations are provided in Section S.4.1 of the Supplementary Information. At the cradle-to-gate phase, the ANN model predicts a GWP of 1.902 kg CO_2_-eq/kgchem compared to 2.080 kg CO_2_-eq/kgchem from SimaPro. At the gate-to-gate phase, the power law regression model predicts a GWP of 0.452 kg CO_2_-eq/kgchem compared to 0.495 kg CO_2_-eq/kgchem from SimaPro. The total GWP predicted by the framework is 2.354 kg CO_2_-eq/kgchem compared to 2.575 kg CO_2_-eq/kgchem from SimaPro. This level of agreement is particularly promising for an early-stage framework, where only limited chemical, thermodynamic, and process information is available. An overall error of 8.6% suggests that the framework is able to capture the dominant contributors to GWP with good accuracy, making it a useful tool for preliminary environmental impact screening.

**Table 5:** Comparison of predicted and SimaPro calculated GWP for isopropanol recovery from a pharmaceutical waste stream

GWP (kg CO_2_-eq/kg chem)	GWP (Framework) (kg CO_2_-eq/kg chem)	GWP (SimaPro) (kg CO_2_-eq/kg chem)
Cradle-to-gate (ANN)	1.902	2.080
Gate-to-Gate (Regression)	0.45156	0.495
Total GWP	2.3536	2.575

#### Case study 2: Ethanol–water hybrid separation systems

In this case study, we evaluate the overall GWP for 1 kg of ethanol produced from the ethanol–water separation system and compare the framework predictions against the climate change impacts reported by Do Thi and Tóth. It is important to note that reproducing the exact absolute values of a reference LCA study is not the objective of this framework. This is not a limitation unique to the proposed approach, as demonstrated in section S5 of the Supplementary Information, an independent reproduction of the reference system using SimaPro, a commercial LCA tool, also yields different absolute GWP values from the reference study. This reflects the well-known sensitivity of LCA results to background LCI database assumptions, which vary across studies and tools even when the same physical system is being evaluated.

**Fig. 12:**
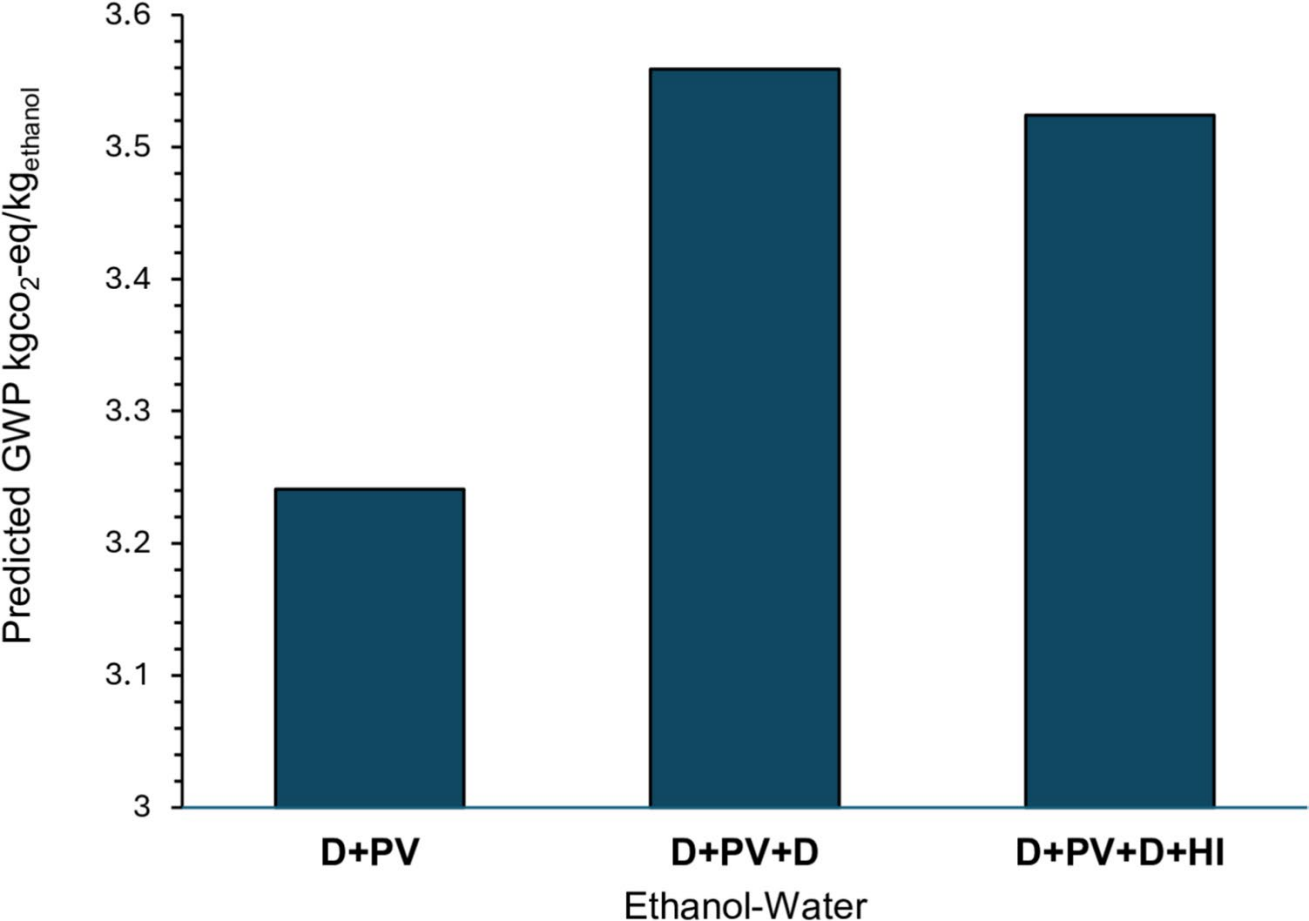
Predicted overall GWP for the three-hybrid ethanol–water separation configurations estimated by the proposed framework

Table 6 presents the overall GWP predicted by the proposed framework for the three configurations. The cradle-to-gate GWP is identical across all three configurations at 3.102 kg CO_2_-eq/kgchem, reflecting that the same chemical system underlies all cases and that production phase impact is independent of the downstream separation configuration. The differences in overall GWP arise entirely from the gate-to-gate term, with D+PV yielding the lowest overall GWP of 3.241 kg CO_2_-eq/kgethanol, followed by D+PV+D+HI at 3.524 kg CO_2_-eq/kgethanol and D+PV+D at 3.559 kg CO_2_-eq/kgethanol as shown in Fig. 12. Detailed calculations are provided in Section S.4.2 of the Supplementary Information.

**Table 6:** GWP for all three scenarios of ethanol–water mixture case study

Scenario	GWP (ANN) (kg CO_2_-eq/kg chem)	GWP (Regression) (kg CO_2_-eq/kg chem)	Overall GWP (kg CO_2_-eq/kg chem)
D+PV	3.102	0.1395	3.2413
D+PV+D	3.102	0.456995	3.558995
D+PV+D+HI	3.102	0.4222	3.5242

The reference study reports climate change values of approximately 0.431, 0.901, and 0.781 kg CO_2_-eq per kg of 99.9 wt.% alcohol product for D+PV, D+PV+D, and D+PV+D+HI, respectively, lower than the framework predictions. However, the rank ordering of configurations is identical between the proposed framework and the reference study, with D+PV yielding the lowest GWP in both cases. This consistent ordering confirms that the framework reliably identifies the least environmentally burdensome configuration, which is its primary intended purpose at early design stages where detailed inventory data are not yet available.

## Conclusion

In this work, we presented an early-stage predictive framework for life cycle environmental impact assessment of chemicals and processes when detailed life cycle inventory (LCI) data and commercial LCA software are unavailable. The approach combines machine learning (ML) algorithms, specifically Artificial Neural Networks (ANN) and eXtreme Gradient Boosting (XGBoost), trained on a combined set of molecular descriptors and thermodynamic properties for ~350 chemicals to predict four cradle-to-gate endpoint metrics: Human Health Impact (HHI), Ecosystem Quality Impact (EQI), Global Warming Potential (GWP), and Resource Utilization Impact (RUI). In addition, a power law regression model is developed to estimate gate-to-gate use-phase GWP as a function of process rate and energy consumption. A Shapley additive explanations (SHAP)-based feature importance analysis confirms that both molecular descriptor and thermodynamic property features contribute meaningfully across all the four impact metrics, indicating that chemical structure and thermodynamic behavior jointly influence environmental impact predictions.

To assess the accuracy and generalizability of the framework, two case studies were conducted. The first involved the estimation of GWP for an isopropanol (IPA) recovery system from a pharmaceutical waste stream, where the framework yielded a total GWP prediction within 8.6% of SimaPro calculated values. The second involved three hybrid ethanol–water separation configurations, where the framework correctly reproduced the rank ordering in terms of the GWP, consistent with published life cycle assessment results and an independent SimaPro reproduction. The hybrid distillation–pervaporation (D+PV) configuration showed the lowest GWP, the heat-integrated D+PV+D+HI configuration exhibited intermediate impacts, and the D+PV+D configuration had the highest GWP. The strong performance across both case studies, particularly given that the framework operates without detailed life cycle inventory data, demonstrates its reliability as a practical tool for early-stage environmental screening of chemicals and processes.

## Supplementary Information

Below is the link to the electronic supplementary material.

Supplementary file1 (PDF 736 KB)

## Data Availability

Data will be made available on request.
