# Supplementary material for: Early-stage environmental impact forecasting of chemicals and processes with machine learning and data analytics tools
Source: Clean Technol Environ Policy. 2026 Apr 4;28(5):120. doi: 10.1007/s10098-026-03479-8 (PMC13050333; doi:10.1007/s10098-026-03479-8)
Supplement: Supplementary file 1 — Supplementary file1 (PDF 736 KB) [file 10098_2026_3479_MOESM1_ESM.pdf]

SUPPLEMENTARY MATERIALS

Early-Stage Environmental Impact Forecasting of Chemicals and Processes with Machine Learning and Data Analytics Tools

Harriet Dufie Appiah, Mathew Conway, Jahnvi Patel, Marcella McMahon, Robert Hesketh, and Kirti M. Yenkie*
Department of Chemical Engineering, Rowan University, Glassboro, NJ, USA
Corresponding author email: yenkie@rowan.edu

S.1. Sequential Backward Feature Selection (SBFS)

The initial feature space contained 200 molecular descriptors and 23 thermodynamic properties. SMILES strings for each chemical were obtained using CIRpy (v1.0.2), then passed to the chemicals (v1.14) and thermo (v0.2.26) libraries to retrieve thermodynamic data, while RDKit (v2023.3.3) was used to compute the molecular descriptors. Given the large number of available features, it is important to retain only those that contribute most to model performance therefore we used the Sequential Backward Feature Selection with linear regression and 5-fold cross-validated mean squared error to select five features from each feature set.

Mathematically, the SBFS procedure can be written as follows:

Input:

F – set of all features

J – evaluation function (5-fold cross-validated mean squared error)

K – desired number of features (= 5)

Initialize:

$t = 0$

$S^{(0)} = F$

While $\left|S^{(t)}\right| > K$:

For each feature $f \in S^{(t)}$:

$\mathrm{score}(f) = J\left(S^{(t)} \backslash \{f\}\right)$

$f^* = \mathrm{argmin}\,\mathrm{score}(f)$

$S^{(t+1)} = S^{(t)} \backslash f^* S^{(t+1)}$

Output:

$S^{(t)}$ with $\left|S^{(t)}\right| = K$

S.2. Machine learning hyperparameter tuning

The performance of the ANN model depends strongly on a set of hyperparameters, such as the number of hidden layers, number of neurons per layer, activation functions, learning rate, dropout rate, loss function, and batch size. In this work, the Adam optimizer was employed to train the ANN, as it uses adaptive learning rates and momentum, which typically lead to faster and more stable convergence compared to standard gradient descent for this type of dataset. The available data was split into training, testing and validation

sets, and the tuning of the hyperparameters was conducted using the validation set by selecting the configuration that yielded the lowest validation error.

Table S1 Hyperparameter tuning for ANN models

Hyperparameter	GWP	RUI	HHI	EQI
Input layer activation function	elu	elu	tanh	elu
Input layer #neurons	200	200	140	200
Input layer dropout rate	0.2631	0.2631	0.1834	0.2631
Input layer initializer	GlorotUniform	GlorotUniform	GlorotUniform	GlorotUniform
Number of hidden layers	5	5	4	5
Hidden layer initializer	GlorotUniform, GlorotUniform, GlorotUniform, GlorotUniform, GlorotUniform	GlorotUniform, GlorotUniform, GlorotUniform, GlorotUniform, GlorotUniform	HeNormal, HeNormal, HeNormal, HeNormal	GlorotUniform, GlorotUniform, GlorotUniform, GlorotUniform, GlorotUniform
Hidden layer #neurons	200, 200, 200, 200, 200	200, 200, 200, 200, 200	250, 250, 250, 250	200, 200, 200, 200, 200
Hidden layer activation function	leaky_relu(alpha=0.051), leaky_relu(alpha=0.051), leaky_relu(alpha=0.051), leaky_relu(alpha=0.051), leaky_relu(alpha=0.051),	leaky_relu(alpha=0.051), leaky_relu(alpha=0.051), leaky_relu(alpha=0.051), leaky_relu(alpha=0.051), leaky_relu(alpha=0.051),	relu, relu, relu, relu	leaky_relu(alpha=0.051), leaky_relu(alpha=0.051), leaky_relu(alpha=0.051), leaky_relu(alpha=0.051), leaky_relu(alpha=0.051),
Hidden layer dropout rate	0.3055, 0.3055, 0.3055, 0.3055, 0.3055,	0.3055, 0.3055, 0.3055, 0.3055, 0.3055,	0.3055, 0.3055,	0.3055, 0.3055,
Output layer activation function	relu	relu	relu	relu
Loss function	Mean squared logarithmic error	Mean squared logarithmic error	Mean squared logarithmic error	Mean squared logarithmic error
Learning rate	0.00108	0.00108	0.00127	0.00108

S.3. Data points for the Technologies used at Gate-to-Gate phase of the Case Studies

This phase leverages a power-law regression correlation to develop regression models that estimate the GWP of the different technologies. The regression equation inspired by cost estimation equation is written as:

$$\left(\frac{\mathrm{GWP_{new}}}{\mathrm{GWP_{ref}}}\right) = \left(\frac{\mathrm{F_{new}}}{\mathrm{F_{ref}}}\right)^{\alpha} \left(\frac{\mathrm{E_{new}}}{\mathrm{E_{ref}}}\right)^{\beta} \quad \text{(S1)}$$

where F denotes the throughput and E denotes the energy consumption of the technology, and $\alpha$and β are regression coefficients which tell if the GWP of the technology is dependent on the feed throughput or energy consumption respectively.

Linearized form:

$$\ln \mathrm{Y} = \alpha \ln \mathrm{X1} + \beta \mathrm{lnX2} \quad \text{(S2)}$$

Where;

$$\mathbf{X1} = \frac{\mathrm{F_{new}}}{\mathrm{F_{ref}}},\ \mathbf{X2} = \frac{\mathrm{E_{new}}}{\mathrm{E_{ref}}},\ \text{and } \mathbf{Y} = \frac{\mathrm{GWP_{new}}}{\mathrm{GWP_{ref}}}$$

Table S2 and **Table S4** present the literature-derived data points used to generate the distillation and pervaporation graphs shown Fig. 11 in the manuscript. In each case, a reference data point with the highest capacity was chosen to normalize the full dataset, and the resulting normalized points were plotted and fitted to obtain the regression coefficients $\alpha$and β.

Table S2 Data points for distillation obtained from literature

	**Feed Rate (kg/s)**	**Energy (kW)**	**GWP (kg CO2-eq/kgchem)**
Chea et al. [1]	0.2778	1175.3	0.2723
Do Thi and Toth. [2]	0.2778	223.3	0.0706
Caballero-Sanchez et al. [3]	0.0130	20.1	0.1295
Cavanagh et al. [4]	0.1261	407.2	0.2067
Cavanagh et al. [4]	0.4856	324.7	0.0500
Cavanagh et al. [4]	0.3089	1022.9	0.2219
Arenas-Grimaldo et al.[5]	0.21	2263	1.94
Arenas-Grimaldo et al.[5]	1.24	5475	0.793
Arenas-Grimaldo et al.[5]	1.45	51360	6.35

Reference data:

Arenas-Grimaldo et al.[5]	1.45	51360	6.35

Table S3 Normalized dataset used to generate the distillation graph

Data	X_1	X_2	Y
1	0.191570881	0.0228831	0.042879454
2	0.191570881	0.004348151	0.011111374
3	0.008939176	0.000391951	0.02038985
4	0.08697318	0.007927391	0.032549324
5	0.3348659	0.006322459	0.007869211
6	0.21302682	0.019917136	0.034939729
7	0.144827586	0.044061526	0.305511811
8	0.855172414	0.106600467	0.12488189
9	1	1	1

After normalization using equation (S2), provided in the Supplementary Information, the coefficients α and β are obtained. These coefficients are then substituted back into equation (S1) to estimate the GWP of any technology at a given process rate and energy consumption. Equation (S3), shows the equation used to generate the GWP of any new data points for distillation

Power-law model for the distillation unit:

$$\left(\frac{\mathrm{GWP_{new}}}{6.35}\right) = \left(\frac{\mathrm{F_{new}}}{1.45}\right)^{0}\left(\frac{\mathrm{E_{new}}}{51360}\right)^{0.702} \quad \text{(S3)}$$

The same procedure described above for distillation applies to pervaporation, using Meng et al. as the reference data.

Table S4 Data points for pervaporation obtained from literature

	**Flux ($L/m^2.h$)**	**Energy (kJ/h)**	**GWP (kg CO_2-eq/kg chem)**
Meng et al.[6]	4.3357	1.22E+07	1.46E-02
Lee et al.[7]	1.3870	2.05E+05	5.500E-03
Norkobilov et al.[8]	0.262	1.54E+06	7.420E-03
Norkobilov et al.[8]	0.2876	6.16E+05	3.160E-03
Do Thi and Toth[2]	1.3000	1.66E+05	1.320E-03

Reference data:

Meng et al.[6]	4.3357	1.22E+07	1.46E-02

Table S5 Normalized dataset used to generate the pervaporation graph

Data	X_1	X_2	Y
1	1	1	1
2	0.3199	0.0167	0.3767
3	0.0604	0.1256	0.5082
4	0.0663	0.0504	0.2164
5	0.2998	0.0135	0.0904

Power-law model for the pervaporation unit:

$$\left(\frac{GWP_{new}}{0.0146}\right) = \left(\frac{F_{new}}{4.3357}\right)^{0.0154} \left(\frac{E_{new}}{1.22*10^7}\right)^{0.411} \tag{S4}$$

S.4. Detailed Calculations of the GWP for the Various Case Studies

S.4.1. Case Study 1: Isopropanol Recovery from a Pharmaceutical Waste Stream

The material and energy balance data for this case study were obtained from the GAMS optimization model provided by Chea et al. [24] and Aspen Plus. Table S6 presents key process parameters for the distillation and pervaporation technologies, including feed flow rate, stream compositions, reboiler and condenser duties for distillation, and feed flux and energy consumption for pervaporation.

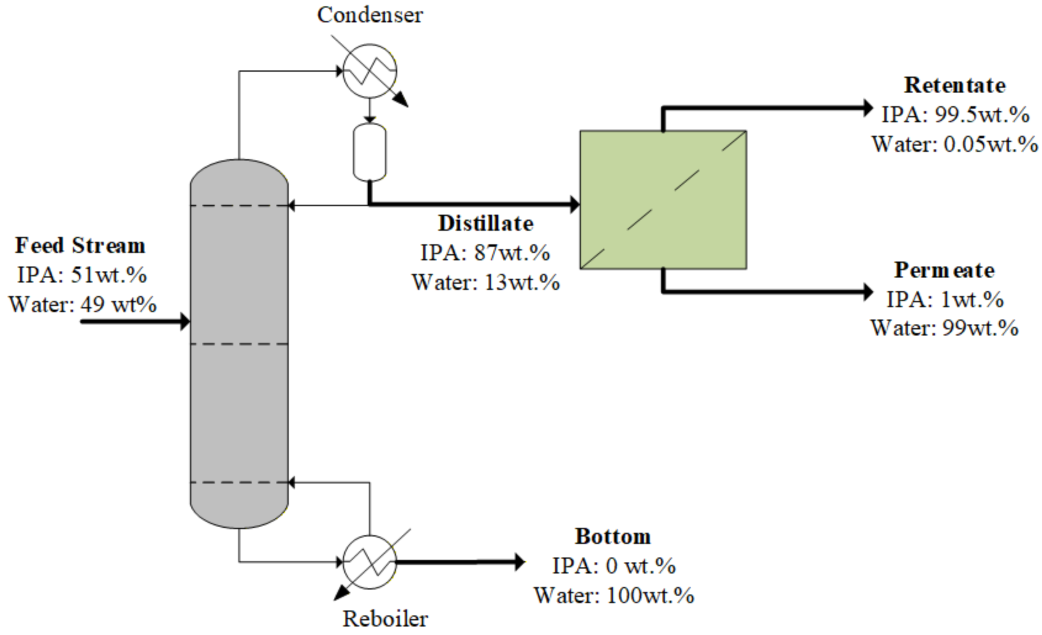

Table S6 Material and energy balance data for the distillation-pervaporation pathway framework for Case Study 1

Technology	Parameter	Value	Unit
**Distillation**	Feed Mass Flow	0.278	kg/s.
	Isopropanol mass fraction	51	%
	Water mass fraction	49	%
	Reboiler duty	2189	MJ/hr.
	Condenser duty	-2042	MJ/hr.
	Total energy	1175.28	kW
**Pervaporation**	Feed Flux	55	$L/m^2.h$
	Total energy	395.313	MJ/hr.

Using equation (S3) to estimate the GWP for distillation in case study 1:

$$\left(\frac{\mathrm{GWP_{new}}}{6.35}\right)=\left(\frac{0.278}{1.45}\right)^{0}\left(\frac{1175.28}{51360}\right)^{0.702}$$

$\mathrm{GWP_{new}}$ = 0.4478kg CO_{2}-eq

Using equation (S4) to estimate the GWP for pervaporation in case study 1:

$$\left(\frac{\mathrm{GWP_{new}}}{0.0146}\right)=\left(\frac{55}{4.3357}\right)^{0.0154}\left(\frac{0.0408*10^{7}}{1.22*10^{7}}\right)^{0.411}$$

$\mathrm{GWP_{new}}$ 0.00376 kg CO_{2}-eq

Table S7 Gate-to-gate GWP for the distillation and pervaporation units in Case Study 1.

**Technology**	**GWP (kg CO_2-eq/kg chem)**
Distillation	0.4478
Pervaporation	0.00376
**Total**	**0.45156**

S.4.2. Case Study 2: Ethanol-Water Hybrid Separation Systems

In the case study presented by Do Thi and Toth [2], the authors examine the recovery of high-purity ethanol and isobutanol from dilute alcohol–water waste streams using hybrid distillation–pervaporation processes. They evaluate the environmental impacts of the different separation configurations, including climate change, alongside their process performance. In our subsequent calculations, we adopt their ethanol-water mixture as the reference case and estimate the GWP of all scenarios using our model, then compare these values with the GWPs reported in their study.

1. **Detailed Gate-to-gate GWP calculation for configuration 1 (D+PV):**

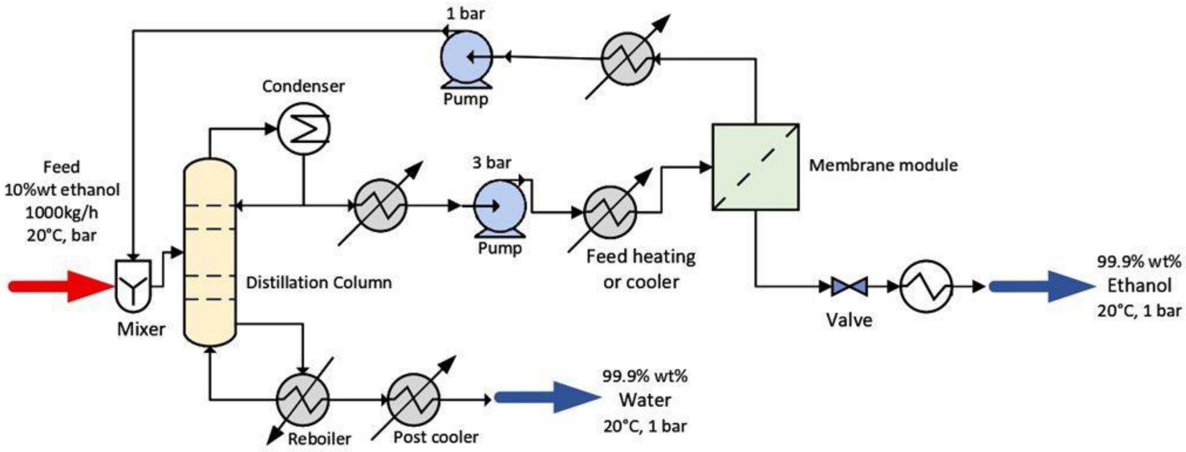

Fig. S1 Process flow diagram of D+PV system

Table S8 Material and energy balance data for the distillation-pervaporation(D+PV) pathway framework for Case Study 2

Technology	Parameter	Value	Unit
**Distillation**	Feed Mass Flow	0.278	kg/s.
	Ethanol mass fraction	10	%
	Water mass fraction	90	%
	Reboiler duty	554.64	MJ/hr.
	Condenser duty	-234.58	MJ/hr.
	Total energy	219.23	kW
**Pervaporation**	Feed Flux	0.07238	L/m^2.h
	Retentate heating	35.58	MJ/hr.
	Permeate cooling	-38.54	MJ/hr.
	Total energy	74.12	MJ/hr.
	Total membrane area	200	m^2

Using equation (S3) to estimate the GWP for distillation in configuration 1in case study 2:

$$\left(\frac{GWP_{new}}{6.35}\right) = \left(\frac{0.278}{1.45}\right)^{0} \left(\frac{219.23}{51360}\right)^{0.702}$$

GWP_{new} = 0.1378 kg CO_2-eq

Using equation (S4) to estimate the GWP for pervaporation in configuration 1 in case study 2:

$$\left(\frac{GWP_{new}}{0.0146}\right) = \left(\frac{0.0728}{4.3357}\right)^{0.0154} \left(\frac{74.12 * 10^{3}}{1.22 * 10^{7}}\right)^{0.411}$$

GWP_{new} = 0.016829 kg CO_2-eq

Table S9 Gate-to-gate GWP for the distillation-pervaporation(D+PV) configuration in Case Study 2

Technology	GWP<br>(kg CO_2-eq/kg chem)
Distillation	0.1378
Pervaporation	0.0016829
**Total**	**0.1395**

2. Detailed Gate-to-gate GWP calculation for configuration 2 (D+PV+D):

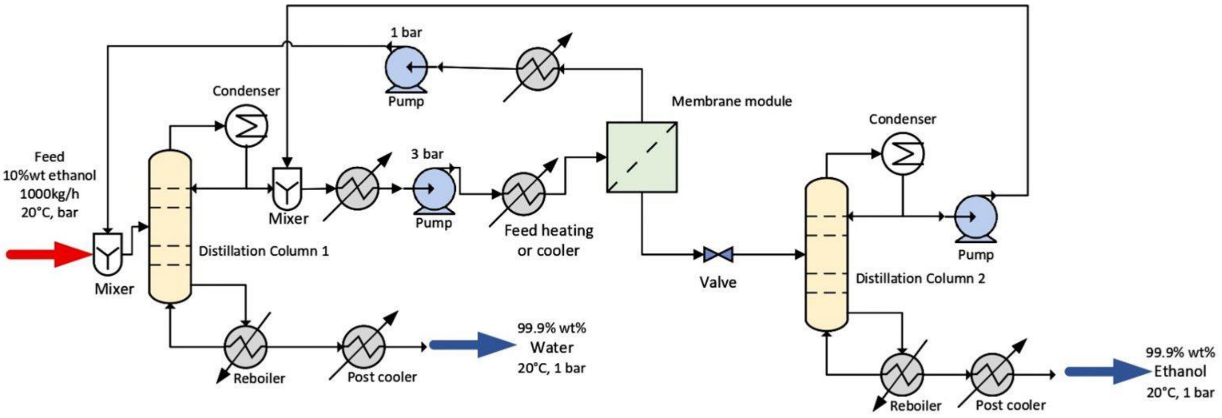

Fig. S2 Process flow diagram of D+PV+D system

Table S10 Material and energy balance data for the distillation-pervaporation-distillation (D+PV+D) pathway framework for Case Study 2

Technology	Parameter	Value	Unit
**1st Distillation**	Feed Mass Flow	0.278	kg/s.
	Ethanol mass fraction	10	%
	Water mass fraction	90	%
	Reboiler duty	713.56	MJ/hr.
	Condenser duty	-396.13	MJ/hr.
	Total energy	308.25	kW
**Pervaporation**	Feed Flux	0.04915	$L/m^2.h$
	Retentate heating	14.86	MJ/hr.
	Permeate cooling	-17.86	MJ/hr.
	Total energy	32.72	MJ/hr.
	Total membrane area	130	m^2
**2nd Distillation**	Feed Mass Flow	0.02764	kg/s.
	Retentate heating	1080.19	MJ/hr.
	Permeate cooling	-1095.41	MJ/hr.
	Total energy	604.33	kW

Using equation (S3) to estimate the GWP for distillation in configuration 2 in case study 2:

$$\left(\frac{\mathrm{GWP_{new}}}{6.35}\right) = \left(\frac{0.278}{1.45}\right)^{0} \left(\frac{308.25}{51360}\right)^{0.702}$$

$\mathrm{GWP_{new}} = 0.1750$ kg CO_2-eq

Using equation (S4) to estimate the GWP for pervaporation in configuration 2 in case study 2:

$$\left(\frac{\mathrm{GWP_{new}}}{0.0146}\right) = \left(\frac{0.04915}{4.3357}\right)^{0.0154} \left(\frac{32.72 * 10^{3}}{1.22 * 10^{7}}\right)^{0.411}$$

$\mathrm{GWP_{new}} = 0.001195$ kg CO_2-eq

Using equation (S3) to estimate the GWP for distillation in configuration 2 in case study 2:

$$\left(\frac{GWP_{new}}{6.35}\right) = \left(\frac{0.02764}{1.45}\right)^{0} \left(\frac{604.33}{51360}\right)^{0.702}$$

GWP_{new} =0.2808 kg CO_{2}-eq

Table S11 Gate-to-gate GWP for the distillation-pervaporation-distillation(D+PV+D) configuration in Case Study 2

**Technology**	**GWP (kg CO_2-eq/kg chem)**
Distillation Column 1	0.1750
Pervaporation	0.001195
Distillation Column 2	10.2808
**Total**	**0.456995**

3. Detailed Gate-to-gate GWP calculation for configuration 3 (D+PV+D+HI):

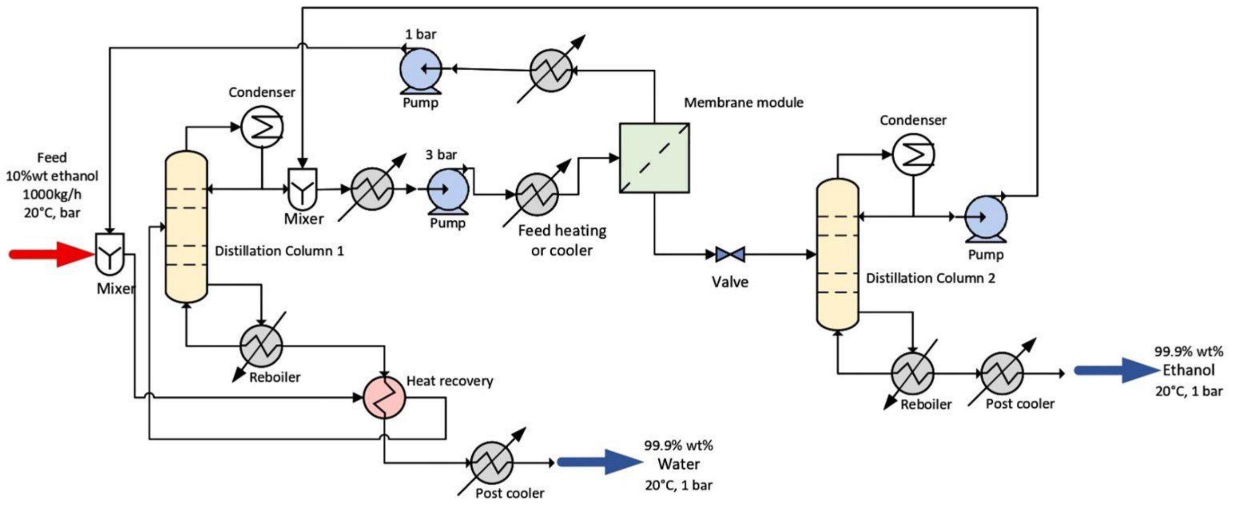

Fig. S3 Process flow diagram of D+PV +D+HI system

Table S12 Material and energy balance data for the distillation-pervaporation-distillation-Heat Integration (D+PV+D+HI) pathway framework for Case Study 2

**Technology**	**Parameter**	**Value**	**Unit**
**1st Distillation**	Feed Mass Flow	0.278	Kg/s.
	Ethanol mass fraction	10	%
	Water mass fraction	90	%
	Reboiler duty	414.49	MJ/hr.
	Condenser duty	-396.87	MJ/hr.
	Total energy	225.38	kW
**Pervaporation**	Feed Flux	0.049397	$L/m^2.h$
	Retentate heating	15.02	MJ/hr.
	Permeate cooling	-18.09	MJ/hr.

	Total energy	33.11	MJ/hr.
	Total membrane area	130	m^2
**2nd Distillation**	Feed Mass Flow	0.027772	Kg/s.
	Retentate heating	1078.65	MJ/hr.
	Permeate cooling	-1093.86	MJ/hr.

Using equation (S3) to estimate the GWP for distillation in configuration 3 in case study 2:

$$\left(\frac{\mathrm{GWP_{new}}}{6.35}\right) = \left(\frac{0.278}{1.45}\right)^{0} \left(\frac{225.38}{51360}\right)^{0.702}$$

$\mathrm{GWP_{new}}$ = 0.1405 kg CO_2-eq

Using equation (S4) to estimate the GWP for pervaporation in configuration 3 in case study 2:

$$\left(\frac{\mathrm{GWP_{new}}}{0.0146}\right) = \left(\frac{0.049397}{4.3357}\right)^{0.0154} \left(\frac{33.11 * 10^3}{1.22 * 10^7}\right)^{0.411}$$

$\mathrm{GWP_{new}}$ = 0.00120 kg CO_2-eq

Using equation (S3) to estimate the GWP for distillation in configuration 3 in case study 2:

$$\left(\frac{\mathrm{GWP_{new}}}{6.35}\right) = \left(\frac{0.027772}{1.45}\right)^{0} \left(\frac{603.48}{51360}\right)^{0.702}$$

$\mathrm{GWP_{new}}$ =0.2805 kg CO_2-eq

Table S13 Gate-to-gate GWP for the distillation-pervaporation-distillation-Heat Integration (D+PV+D+HI) configuration in Case Study 2

**Technology**	**GWP (kg CO_2-eq/kg chem)**
Distillation Column 1	0.1405
Pervaporation	0.00120
Distillation Column 2	0.2805
**Total**	**0.4222**

Once the GWPs for the production phase have been obtained from the ANN model and those for the use phase from the regression models described above, the overall life-cycle GWP of the process is calculated as:

$$GWP_{overall} = GWP_{production} + GWP_{use-phase}$$

Table S14 GWP for all three scenarios of ethanol-water mixture case study

Scenario	**GWP (ANN) (kg CO_2-eq/kg chem)**	**GWP (Regression) (kg CO_2-eq/kg chem)**	**Overall GWP (kg CO_2-eq/kg chem)**
**D+PV**	3.102	0.1395	3.2413
**D+PV+D**	3.102	0.456995	3.558995
**D+PV+D+HI**	3.102	0.4222	3.5242

Having estimated the GWP for our case study, we next summarize how Do Thi and Toth calculated their climate change values in order to enable a consistent comparison.

S.5. Independent Reproduction of the Do Thi and Tóth Ethanol-Water Separation System Using SimaPro

Using the inventory data reported in Table S15, the Environmental Footprint (EF) 3.1 (adapted) method was applied in SimaPro to quantify the climate change impacts of the three ethanol-water separation configurations, normalized to a functional unit of 1 kg of the ethanol product. The inventory accounted for feed inputs, thermal and auxiliary utility consumption, and process output streams for each configuration. The calculated climate change impacts reported in Table S16 further confirm that while different LCA tools and background databases may yield different absolute impact values for the same system, the rank ordering of configurations is consistently preserved, with D+PV yielding the lowest climate change impact across all approaches considered.

Table S15 Inventory data for the production of 1 kg of ethanol in SimaPro

	**D+PV**	**D+PV+D**	**D+PV+D+HI**
**Feed Streams**			
Ethanol (kg)	100	100	100
Water(kg)	900	900	900
**Utilities**			
Reboiler duty (MJ)	554.64	1793.75	1493.14
Condenser duty (MJ)	234.58	1491.51	1490.73
Retentate heating (MJ)	35.58	14.86	15.02
Permeate cooler (MJ)	38.54	17.86	18.09
**Products**			
Ethanol	99.218	99.404	99.897
Water by-product	900.782	900.596	900.103

Table S16 Results of the Impact Assessment Using the Adapted EF Method

Impact category	Unit	D+PV	D+PV+D	D+PV+D+HI
Climate change	kg CO2 eq	3.020769	5.989985	5.986691
Ozone depletion	kg CFC11 eq	3.25E-07	6.48E-07	6.83E-07
Ionising radiation, HH	kBq U-235 eq	0.126307	0.173651	0.193641
Photochemical ozone formation, HH	kg NMVOC eq	0.009267	0.011011	0.013393
Respiratory inorganics	disease inc.	2.18E-07	2.64E-07	2.81E-07
Non-cancer human health effects	CTUh	5.92E-07	7.07E-07	7.35E-07
Cancer human health effects	CTUh	3.11E-08	3.99E-08	4.47E-08
Acidification terrestrial and freshwater	mol H+ eq	0.020173	0.026325	0.02753
Eutrophication freshwater	kg P eq	0.000886	0.001265	0.001272
Eutrophication marine	kg N eq	0.007192	0.007547	0.008338
Eutrophication terrestrial	mol N eq	0.065426	0.069089	0.077908
Ecotoxicity freshwater	CTUe	6.024338	6.003585	6.626124
Land use	Pt	210.5111	211.8599	217.3075
Water scarcity	m3 depriv.	6.515273	6.882163	6.879051
Resource use, energy carriers	MJ	34.69875	74.53624	75.28004
Resource use, mineral and metals	kg Sb eq	5.23E-06	8.61E-06	9.47E-06
Climate change - fossil	kg CO2 eq	2.769728	5.737684	5.735323
Climate change - biogenic	kg CO2 eq	0.021424	0.022467	0.022496
Climate change - land use and transform.	kg CO2 eq	0.229617	0.229834	0.228872

S.5. 1. Contribution Tree Diagrams with Node Cut-Offs

Also presented below are contribution tree diagrams that highlight the processes making the largest contributions to the overall environmental impact in each scenario. A node cut-off is applied so that only processes contributing above a specified threshold of the total impact are displayed, with cut-off values of 6.6%, 8.69%, and 8.76% applied for the D+PV, D+PV+D, and D+PV+D+HI configurations respectively. The contribution trees are shown in Figures S4, S2, and S3 respectively. Across all three configurations, the dominant contributors to environmental impact are the ethanol feed stream and the thermal utilities, consistent with the energy-intensive nature of distillation-based separation processes. The width of the arrows in the network figures indicates the relative magnitude of each process contribution to the overall impact.

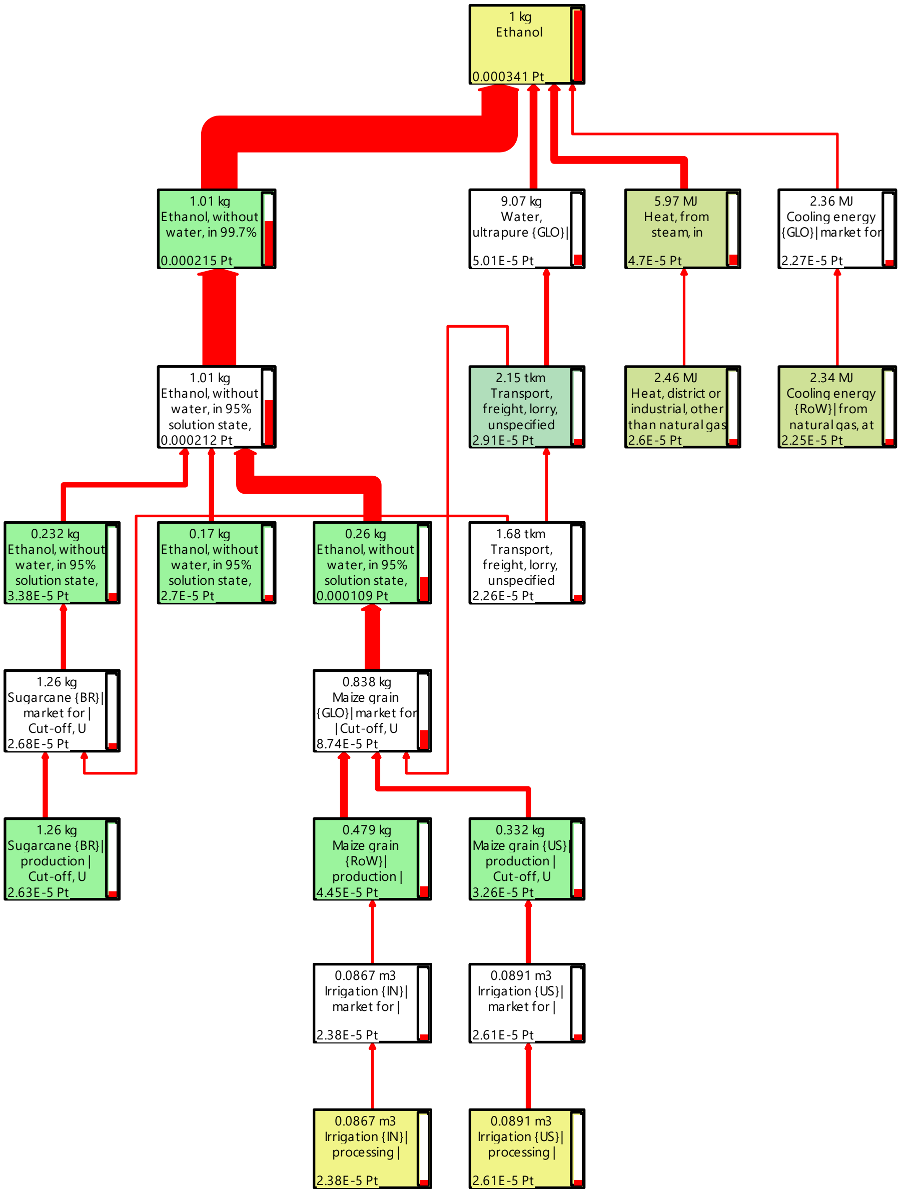

Fig. S4 D+PV: 6.6% node cut-off

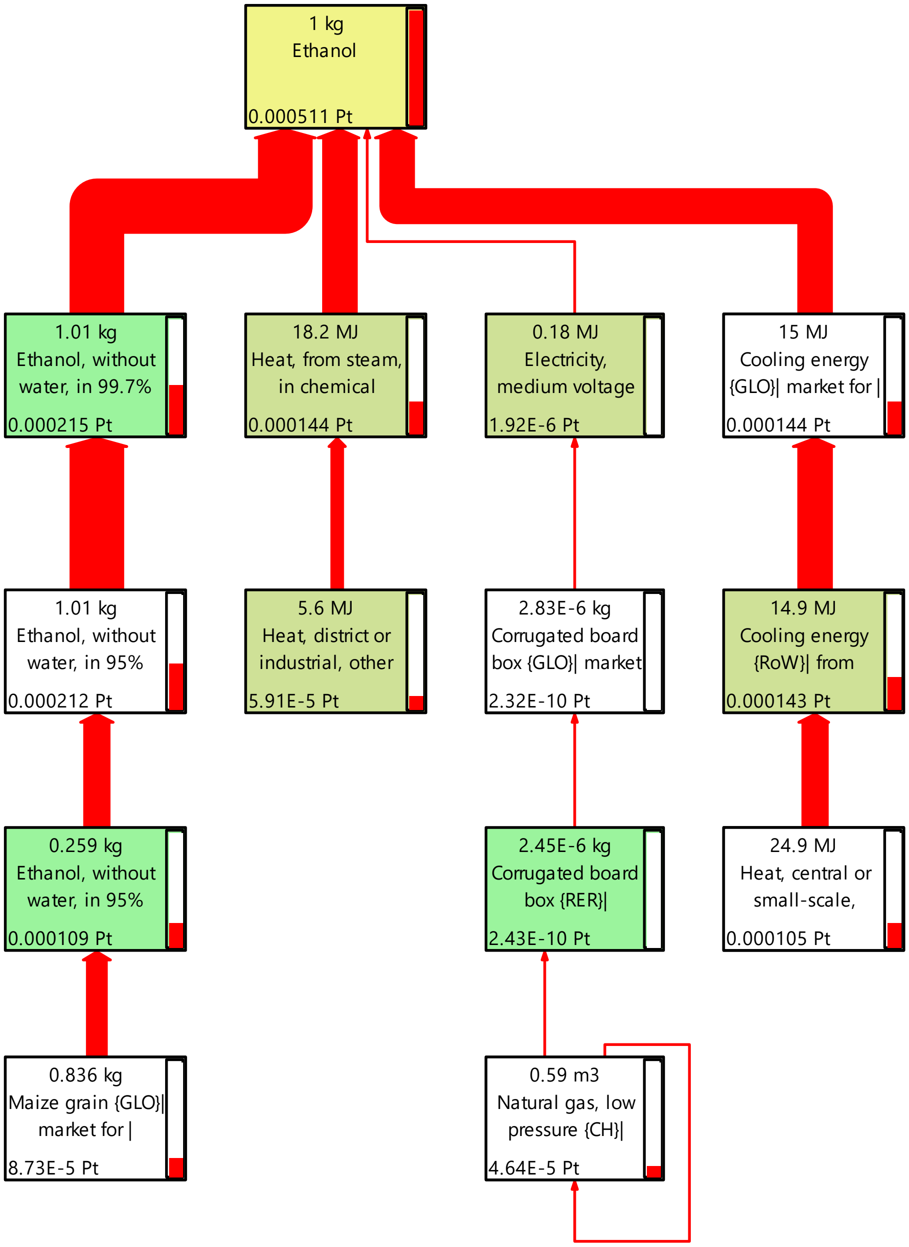

Fig. S5 D+PV+D: 8.69% node cut-off

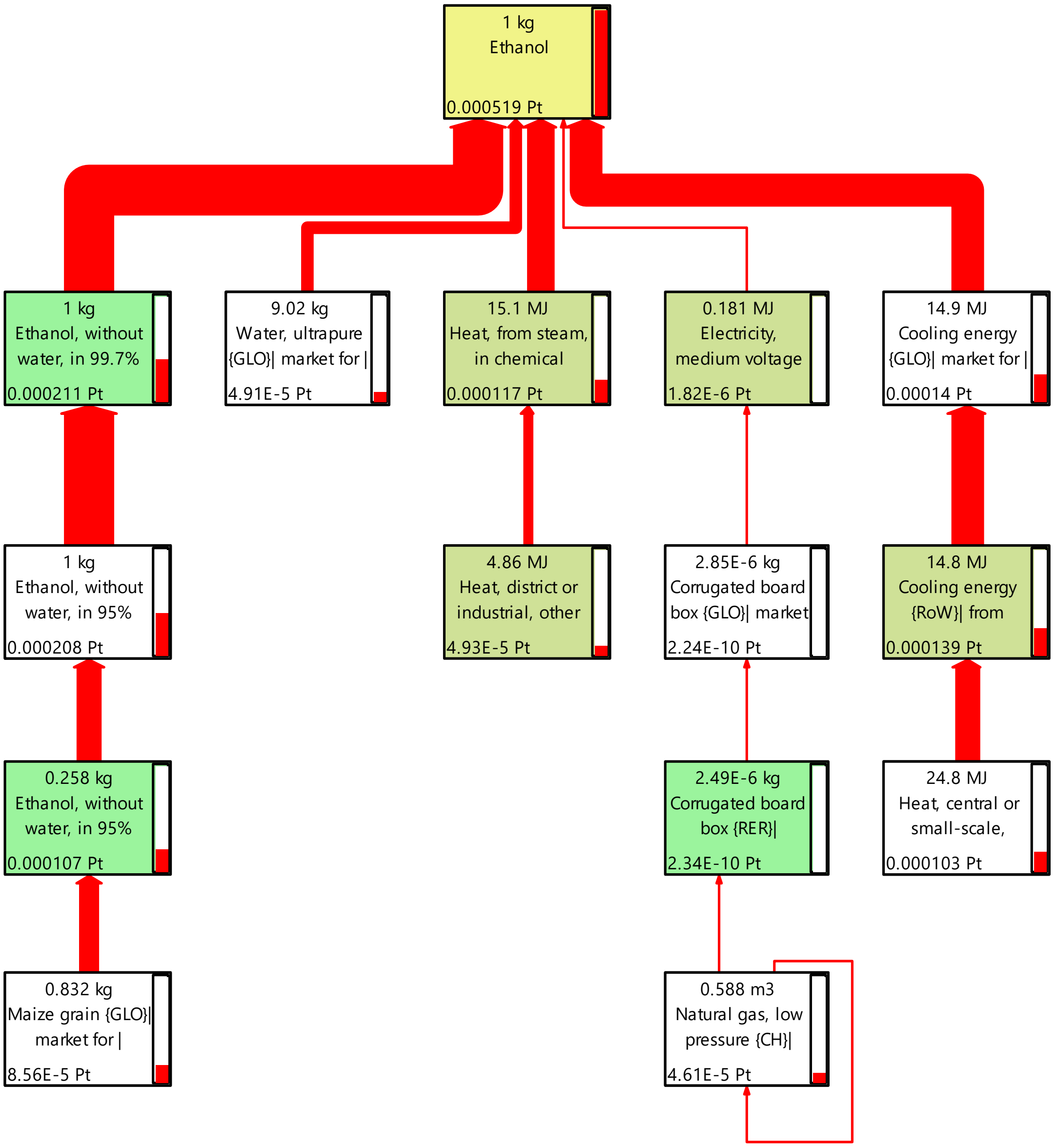

Fig. S6 D+PV+D+HI: 8.76% node cut-off

S.7. Power-Law Scaling Model Plots for Additional Technologies

Table S17 Data points for dryer obtained from literature

	Capacity (m^3/h)	Energy (kJ/h)	GWP (kg CO_2-eq/kg chem)
Iolanda De Marco et al.[9]	1.04E-03	1.47E+02	8.84E+02
Iolanda De Marco et al.[9]	1.00E-02	1.00E+01	1.52E+02
Iolanda De Marco et al.[9]	2.00E-02	8.33E+00	1.29E+02
Iolanda De Marco et al.[9]	4.00E-02	1.57E+01	1.27E+02
Righi et al.[10]	1.08E-03	5.25E+00	1.18E+00
Gésan-Guiziou et al.[11]	3.07E-01	1.60E+03	3.69E+02
Gésan-Guiziou et al.[11]	1.65E+00	8.47E+03	1.95E+03

Reference data:

Gésan-Guiziou et al.[11]	1.65E+00	8.47E+03	1.95E+03

	Flux (L/m^2.h)	Energy (kJ/h)	GWP (kg CO_2-eq/kg chem)
Li et al.[12]	6.71E+01	1.68E+04	2.90E-02
Ribera et al.[13]	2.70E+01	1.48E+06	1.91E+02
O'Connell et al.[14]	2.00E+01	1.25E+05	2.53E+01
Teow et al.[15]	7.08E+01	7.56E+02	1.51E+01
Ribera et al.[16]	1.27E+01	7.56E+02	3.40E-02
Teow et al. [94]	6.20E+01	3.72E+04	4.13E+00

Reference data:

Li et al.[12]	6.71E+01	1.68E+04	2.90E-02

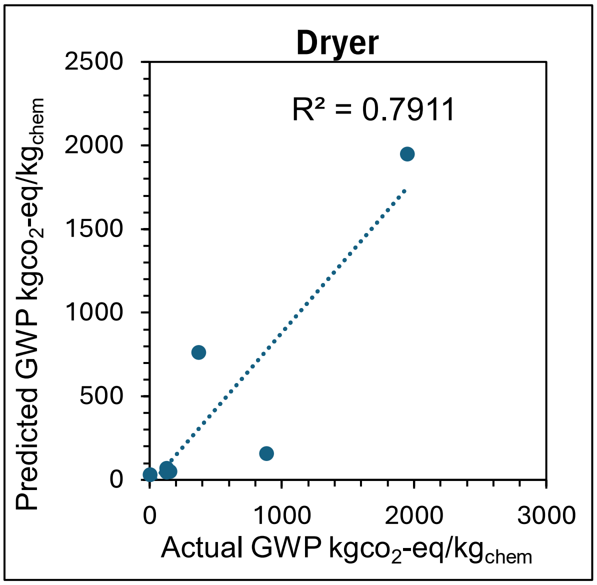

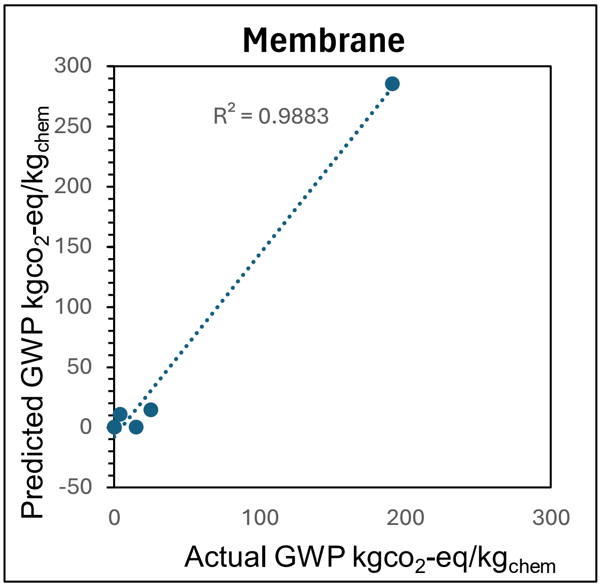

References

[1] J. D. Chea, A. L. Lehr, J. P. Stengel, M. J. Savelski, C. S. Slater, and K. M. Yenkie, "Evaluation of Solvent Recovery Options for Economic Feasibility through a Superstructure-Based Optimization Framework," *Ind Eng Chem Res*, 2020.

[2] H. T. Do Thi and A. J. Toth, "Environmental evaluation and comparison of hybrid separation methods based on distillation and pervaporation for dehydration of binary alcohol mixtures with life cycle, PESTLE, and multi-criteria decision analyses," *Sep. Purif. Technol.*, vol. 348, p. 127684, Nov. 2024, doi: 10.1016/j.seppur.2024.127684.

[3] L. Caballero-Sanchez, A. A. Vargas-Tah, P. E. Lázaro-Mixteco, and A. J. Castro-Montoya, "Recovery of 1,4-butanediol from aqueous solutions through aqueous two-phase systems with K2CO3," *Chem. Eng. Res. Des.*, vol. 201, pp. 150–156, Jan. 2024, doi: 10.1016/j.cherd.2023.11.015.

[4] E. J. Cavanagh, M. J. Savelski, and C. S. Slater, "Optimization of environmental impact reduction and economic feasibility of solvent waste recovery using a new software tool," *Chem. Eng. Res. Des.*, vol. 92, no. 10, pp. 1942–1954, Oct. 2014, doi: 10.1016/j.cherd.2014.02.022.

[5] C. Arenas-Grimaldo, J. G. Avendaño-Guerrero, C. E. Molina-Guerrero, and J. G. Segovia-Hernández, "Design and control of a distillation sequence for the purification of bioethanol obtained from sotol bagasse (Dasylirium sp.)," *Chem. Eng. Res. Des.*, vol. 203, pp. 11–17, Mar. 2024, doi: 10.1016/j.cherd.2023.12.039.

[6] D. Meng *et al.*, "Energy, economic and environmental evaluations for the separation of ethyl acetate/ethanol/water mixture via distillation and pervaporation unit," *Process Saf. Environ. Prot.*, vol. 140, pp. 14–25, Aug. 2020, doi: 10.1016/j.psep.2020.04.039.

[7] H.-Y. Lee, S.-Y. Li, and C.-L. Chen, "Evolutional Design and Control of the Equilibrium-Limited Ethyl Acetate Process via Reactive Distillation−Pervaporation Hybrid

Configuration," *Ind. Eng. Chem. Res.*, vol. 55, no. 32, pp. 8802–8817, Aug. 2016, doi: 10.1021/acs.iecr.6b01358.
[8] A. Norkobilov, D. Gorri, and I. Ortiz, "Process flowsheet analysis of pervaporation-based hybrid processes in the production of ethyl tert-butyl ether," *J. Chem. Technol. Biotechnol.*, vol. 92, no. 6, pp. 1167–1177, Jun. 2017, doi: 10.1002/jctb.5186.
[9] Iolanda De Marco, Stefano Riemma, and Raffaele Iannone, "Lca of aerogel production using supercritical gel drying: from bench scale to industrial scale," *Chem. Eng. Trans.*, vol. 57, pp. 241–246, May 2017, doi: 10.3303/CET1757041.
[10] S. Righi *et al.*, "A life cycle assessment of poly-hydroxybutyrate extraction from microbial biomass using dimethyl carbonate," *J. Clean. Prod.*, vol. 168, pp. 692–707, Dec. 2017, doi: 10.1016/j.jclepro.2017.08.227.
[11] G. Gésan-Guiziou *et al.*, "Life Cycle Assessment of a milk protein fractionation process: Contribution of the production and the cleaning stages at unit process level," *Sep. Purif. Technol.*, vol. 224, pp. 591–610, Oct. 2019, doi: 10.1016/j.seppur.2019.05.008.
[12] B. Li, J. Wu, and J. Lu, "Life cycle assessment considering water-energy nexus for lithium nanofiltration extraction technique," *J. Clean. Prod.*, vol. 261, p. 121152, Jul. 2020, doi: 10.1016/j.jclepro.2020.121152.
[13] G. Ribera, F. Clarens, X. Martínez-Lladó, I. Jubany, V.Martí, and M. Rovira, "Life cycle and human health risk assessments as tools for decision making in the design and implementation of nanofiltration in drinking water treatment plants," *Sci. Total Environ.*, vol. 466–467, pp. 377–386, Jan. 2014, doi: 10.1016/j.scitotenv.2013.06.085.
[14] D. O'Connell, M. Savelski, and C. S. Slater, "Life cycle assessment of dewatering routes for algae derived biodiesel processes," *Clean Technol. Environ. Policy*, vol. 15, no. 4, pp. 567–577, Aug. 2013, doi: 10.1007/s10098-012-0537-7.
[15] Y. H. Teow, M. T. Chong, K. C. Ho, and A. W. Mohammad, "Comparative environmental impact evaluation using life cycle assessment approach: a case study of integrated membrane-filtration system for the treatment of aerobically-digested palm oil mill effluent," *Sustain. Environ. Res.*, vol. 31, no. 1, p. 15, Dec. 2021, doi: 10.1186/s42834-021-00089-5.
[16] G. Ribera, F. Clarens, X. Martínez-Lladó, I. Jubany, V.Martí, and M. Rovira, "Life cycle and human health risk assessments as tools for decision making in the design and implementation of nanofiltration in drinking water treatment plants," *Sci. Total Environ.*, vol. 466–467, pp. 377–386, Jan. 2014, doi: 10.1016/j.scitotenv.2013.06.085.
